# Identification of Microbiome Associations with Tacrolimus Pharmacokinetics in Adult Hematopoietic Cell Transplantation Using Population Pharmacokinetic and Machine Learning

**DOI:** 10.1007/s11095-026-04152-x

**Published:** 2026-07-20

**Authors:** Moataz E. Mohamed, Shen Cheng, Christopher Staley, Armin Rashidi, Najla El Jurdi, Shernan G. Holtan, Pamala A. Jacobson

**Affiliations:** 1 Department of Experimental and Clinical Pharmacology, College of Pharmacy, University of Minnesota, Minneapolis, MN 55455 USA; 2 Department of Surgery, School of Medicine, University of Minnesota, Minneapolis, MN 55455 USA; 3 Clinical Research Division, Fred Hutchinson Cancer Center, Seattle, WA 98195 USA; 4 Immune Deficiency Cellular Therapy Program, Center for Cancer Research (CCR), National Cancer Institute (NCI), National Institutes of Health (NIH), Bethesda, MD 20892 USA; 5 Department of Medicine, Transplant and Cellular Therapy Program, Comprehensive Cancer Center, Roswell Park, Buffalo, NY 14203 USA

**Keywords:** bone marrow transplantation, microbiome, pharmacogenomics, population pharmacokinetics, tacrolimus

## Abstract

**Purpose:**

Tacrolimus (TAC) is known for its high pharmacokinetic variability which cannot be fully explained by pharmacogenomic (PGx) and clinical variables. We identified gut microbiome associated with TAC pharmacokinetic variability in allogeneic hematopoietic cell transplant (HCT) recipients.

**Methods:**

In this observational study, metagenomic shotgun sequencing was used to analyze stool microbiome collected within ± 10 days from time of first oral TAC trough at steady state. TAC steady state concentrations (222 IV continuous infusion and 436 oral troughs) were modeled to estimate TAC clearance (CL) and oral bioavailability (F) using nonlinear mixed effects modeling. The effect of clinical covariates, PGx variants and concomitant medications on CL and F were evaluated. Machine learning was used to identify bacterial species associated with variability in F and CL. The identified species were incorporated into the final model, and simulations were conducted to estimate their clinical relevance on oral TAC troughs.

**Results:**

TAC population CL was 6.91 L/h and population F was 64.4%. TAC CL was increased in those with CYP3A5*1 genotype and reduced with voriconazole use and if estimated glomerular filtration rate < 60 ml/min/1.73 m^2^. TAC F increased with laxative use and decreased with corticosteroid use. *Limosilactobacillus fermentum* had the strongest positive effect on oral TAC troughs while *Bacteroides uniformis* had the strongest negative effect.

**Conclusion:**

Gut microbiome contributes to the inter-patient variability in TAC CL and oral F.

**Supplementary Information:**

The online version contains supplementary material available at https://doi.org/10.1007/s11095-026-04152-x.

## Introduction

Tacrolimus (TAC) is a cornerstone calcineurin inhibitor immunosuppressant used in allogeneic hematopoietic cell transplant (HCT) to prevent immunologic donor-recipient complications such as graft-versus-host disease (GvHD) and to facilitate engraftment of the donor cells [1]. TAC has a narrow therapeutic index which necessitates therapeutic drug monitoring (TDM) of blood concentrations to maintain a therapeutic range and guide dosing adjustments [2]. Low TAC exposure (< 5 ng/mL) in allogeneic HCT has been associated with a higher risk of GvHD [3], while high TAC concentrations increase the risk of toxicities [4].

TAC undergoes extensive first pass metabolism due to intestinal and hepatic cytochrome P450 (CYP) 3A4 and 3A5 enzymes [5]. It is primarily absorbed in the small intestine however evidence shows it can also be absorbed throughout the colon [6]. TAC is passively absorbed into the intestinal enterocytes where it is extensively pumped back into the intestinal lumen by P-glycoprotein protein (PGP), an ATP-dependent efflux transporter, thereby limiting absorption.

The association between *CYP3A5* and *3A4* genetic variants and TAC pharmacokinetics is well established and significantly alters its pharmacokinetics [7]. In kidney transplantation, clinical factors with the genetic variants (CYP3A5*3, *6,*7 and CYP3A4*22) explain 40.1% of TAC pharmacokinetic variability in those of European ancestry [8], and 53.9% in African American ancestry [9], suggesting that additional yet uncharacterized factors may contribute to this variability. Other pharmacogenomic variants may impact TAC pharmacokinetics such as the ATP-binding cassette B1 (ABCB1, encoding for PGP transporter) and P450 oxidoreductase (POR) genes but the evidence remains mixed [10].

Alterations in the gut microbiome are known to influence metabolism and absorption of numerous drugs and the impact of the gut microbiome on TAC has recently been studied in small numbers of patients after solid organ transplant and there are no data in HCT [11]. Changes in gut microbiome composition and dysbiosis are common, and can be profound after HCT due to conditioning regimens which include chemotherapy and radiation, immunosuppressant use, mucosal damage, dietary changes, and antibiotic use [12, 13]. Gut microbiome may also influence TAC pharmacokinetics through direct and indirect interactions [11]. The first human study to find an association between TAC pharmacokinetics and stool microbiome was conducted on a small cohort of kidney transplant recipients where *Faecalibacterium prausnitzii* abundance in stool was significantly associated with higher TAC dose requirements [14]. The same group found that TAC is metabolized to a less active bacterial formed TAC metabolite (C-9 keto reduction metabolite) when incubated with *Faecalibacterium prausnitzii* and other bacteria, primarily from the Clostridiales order [15]. This bacterial formed TAC C-9 keto metabolite was also present in blood of kidney transplant recipients after oral TAC administration [15]. Other evidence of association between stool microbiome and TAC was reported in heart, liver and kidney transplantation [11]. *In vitro* evidence also supports metabolism of TAC by commonly prevalent intestinal bacteria [16, 17]. Gut microbiota and their metabolites can indirectly influence TAC pharmacokinetics through regulation of host drug metabolizing and transporter enzymes. Studies in mice and rats show that the gut microbiome and/or bacterial formed metabolites influence the expression and activity of hepatic *CYP450* enzymes [18, 19] as well as *ABCB1* regulation [20].

It is crucial to understand the sources of TAC pharmacokinetic variability to improve immunosuppressive outcomes and reduce TAC adverse effects in HCT. Understanding the interactions between TAC pharmacokinetics, pharmacogenomics and gut microbiome will better explain sources of exposure variability resulting in improved TAC dose individualization. To the best of our knowledge, this is the first study to investigate the associations between TAC pharmacokinetics and gut microbiome in HCT. This study incorporated a novel approach of combined machine learning with population pharmacokinetic modeling to identify gut bacterial species that influence TAC pharmacokinetic variability while accounting for pharmacogenomic and clinical factors. The main study aim was to identify stool bacterial species associated with TAC clearance (CL) and oral bioavailability (F) and to determine if these effects resulted in clinically meaningful changes in TAC pharmacokinetics. .

## Materials and Methods

### Study Design and Patient Selection

This is an observational microbiome and pharmacogenomic study using samples obtained from adult participants enrolled in the “Monitoring of Immune and Microbial Reconstitution in Hematopoietic Cell Transplantation and Novel Immunotherapies” study (MT2017-12). The study was approved by the University of Minnesota Institutional Review Board (STUDY00000802) and informed consent was obtained from all participants. The study was performed in accordance with the Declaration of Helsinki.

Adult participants (≥ 18 years) enrolled in the parent study were included in the current study if they 1) received an allogeneic HCT, 2) received continuous intravenous (IV) infusion TAC beginning at time of transplant followed by immediate release oral TAC capsules as a part of the immunosuppressive regimen, 3) had TAC trough concentrations measured as part of routine clinical care and available in the electronic medical record, and 4) had a pretransplant blood sample and posttransplant stool samples available. Participants were excluded if they 1) had a history of liver transplant and/or liver dysfunction (total bilirubin > 3 × upper limit of normal) at time of admission for HCT, or 2) had a colostomy or ileostomy.

### Data Collection and Tacrolimus Concentrations and Dosing

Clinical data, TAC steady state concentrations [continuous IV infusion concentrations (C_ss_) and oral troughs (C_t,ss_)] and doses were collected from the electronic medical records. Steady state concentrations were defined as concentrations measured after at least 48 h of unchanged daily doses. As a part of the adult immunosuppression regimen, TAC was initiated on day −3 pre-transplant as an IV continuous infusion at a starting dose of 0.03 mg/kg/day and when tolerated, TAC was converted to oral immediate release formulation dosing, generally around 14 days posttransplant. Target TAC troughs were 12–15 ng/ml in the first week post-HCT, if tolerated, and then 5–10 ng/mL. TAC whole blood concentrations were measured using LC/MS in the clinical drug analysis laboratory, as part of routine clinical transplant care. TAC concentrations were measured 3 times per week for the first week then once weekly while receiving IV continuous infusion. Similarly, after transitioning to oral TAC, trough concentrations were measured 3 times per week until the patient was stable and then once weekly until TAC discontinuation.

### Biospecimen Collection and Storage

Pre-transplant recipient peripheral blood mononuclear cells were collected from blood and stored in vapor phase liquid nitrogen until DNA extraction. Stool samples were collected on day 14 (± 7 days) and day 28 (± 7 days) post-transplant. Stool samples were homogenized using a vortex mixer and stored in RNAlater at −80°C until further processing and bacterial DNA extraction. All samples were stored in the Translational Therapy Lab biorepository prior to analysis.

### Genotyping

Genotyping of the HCT recipient DNA was conducted on a Infinium Global Diversity Array v1.0 (Illumina, CA, USA) using Illumina iScan (Illumina, CA, USA). GenomeStudio (v2.0, Illumina, CA, USA) was used to analyze the genotyping array, create pLink files (v1.90b7.2) and assign genotypes [21]. Genetic variants in *CYP3A5* [CYP3A5*3 (rs776746), CYP3A5*6 (rs10264272), CYP3A5*7 (rs41303343)], *CYP3A4* [CYP3A4*22 (rs35599367), CYP3A4*1B (rs2740574)], *POR* [POR*28 (rs1057868)] and *ABCB1* [*1236C* > *T* (rs1128503)] were obtained from the array and selected for analysis due to their potential associations with TAC pharmacokinetics. All analyzed genetic variants were in Hardy–Weinberg equilibrium.

### Stool Meta-genomic Shotgun Sequencing and Bioinformatics

Among the collected and sequenced stool samples (at day 14 and day 28 post-HCT), we selected the sample that was collected ± 10 days from the first oral TAC steady state trough concentration for analysis which represented the nearest time to IV to oral TAC formulation conversion. DNA from stool samples were extracted using the DNeasy Powerlyzer PowerSoil kit (QIAGEN, Hilden, Germany) in the QIAcube instrument (QIAGEN, Hilden, Germany) according to the manufacturer’s protocol. Metagenomic shotgun sequencing was conducted by the University of Minnesota Genomics Center. Libraries were prepared using Illumina NexteraXT 1/4 reactions and sequencing was performed on Aviti Element at a read length of 2 × 150-bp (Element Biosciences, CA, USA). Metagenomic shotgun sequencing was processed using the biobakery pipeline [22]. The fastq files were preprocessed using kneaddata (v0.12.2) resulting in a median output of ~ 21 million reads and a minimum of 4 million reads per sample and MetaPhlAn (v4.1.1) to construct bacterial relative abundance profiles. Differential abundance analyses were evaluated in a subgroup of patients (*n* = 35) who provided stool samples at both day 14 and day 28, allowing assessment of within-patient changes in the microbiome over time. In addition, abundances in samples collected before or at first steady state TAC oral trough were compared to those collected after first steady state TAC oral trough. The differential abundance analyses were conducted using MaAsLin 3 (version 0.99.11) [23].

### Population Pharmacokinetic Modeling

Tacrolimus steady state concentrations (*n* = 658) from 44 HCT recipients including average steady state concentrations (C_ss_, *n* = 222) during continuous IV infusion and steady state trough concentrations (C_t,ss_, *n* = 436) during oral administration were used for model development. All TAC concentrations up to 60 days after the first oral TAC trough in steady state were included in the modeling. Given that only a single microbiome assessment was available no concentrations after 60-days after first oral TAC trough in steady state were used to minimize the likelihood of meaningful microbiome changes during the observation period. C_ss_ and oral C_t,ss_ were modeled simultaneously using steady state infusion model (Eqs. 1 and 2) to estimate TAC CL (L/hr) and oral F in nonlinear mixed-effects modeling software (NONMEM v7.5, ICON Development Solutions, EllicottCity, Maryland) with Perl-speaks-NONMEM (PsN v5.3.1, Uppsala University, Uppsala,Sweden). A steady state continuous infusion model was used for oral TAC trough concentrations as the drug’s long half-life implies minimal fluctuation at trough, therefore trough concentrations were assumed to approximate the daily average IV steady state concentration [24–26].

1 $$C_{av,ss}=\frac{IV\;infusion\;rate}{Clearance\;\left(CL\right)}$$

2 $$C_{trough,ss}=\frac{Daily\;Dose\;x\;Bioavailability\;(F)}{Clearance\;\left(CL\right)\;\times\;24}$$

The method of First-Order Conditional Estimation with Interaction (FOCE-I) was used for model development, with Pirana (v 23.10.1, build 1, Certara, Princeton, New Jersey) as the modeling interface.

The interindividual variability (IIV) of CL was modeled assuming a log-normal distribution (Eq. 3).

3 $${CL}_{i}=TVCL\times {e}^{{\eta}_{CL,i}}$$

where, CLi is the individual TAC CL, TVCL is the typical value of the CL, and ${\eta}_{CL,i}$ is the individual random effect, which is assumed to be normally distributed with a mean of 0 and variance of ω_(1,1)_^2^.

F was only estimated for oral TAC and was fixed as 1 for IV continuous infusion observations. The IIV in F was estimated assuming a logit normal distribution (Eq. 4), to constrain the individual estimates of bioavailability between 0–1 to reflect the physiological nature of that parameter. Inverse-logit transformation (Eq. 5) was used to convert Logit(F_*i*_) to F_i_.

4 $$Logit\left({F}_{i}\right)=Logit\left(TVF\right)+{\eta}_{F,i}$$

where, Logit(F_i_) is the logit of individual TAC F, Logit(TVF) is the logit of typical F, and ${\eta}_{F,i}$ is the individual random effect assumed to be normally distributed with a mean of 0 and variance of ω_(2,2)_^2^.

An off-diagonal correlation term between ω_(1,1)_^2^ and ω_(2,2)_^2^ was initially included in the base model development but was removed from the subsequent models after introducing covariates to improve stability.

5 $${F}_{i}=\frac{{e}^{Logit({F}_{i})}}{{e}^{Logit({F}_{i})}+1}$$

where, F_i_ is the individual bioavailability.

The proportional, additive and combined residual unexplained variability (RUV) error models were tested to characterize RUV. The proportional error model (Eq. 6) was selected because it adequately characterizes the RUV, as reflected by the objective function value (OFV) and diagnostic plots, while maintaining model parsimony.

6 $${C}_{ij}={C}_{pred,ij}*(1+{\varepsilon}_{prop,ij})$$

### Clinical Factors, Pharmacogenomic, Concomitant Medication and Alpha Diversity Covariate Selection

Clinical factors, genetic variants and concomitant medication covariates were evaluated for their relationship with TAC CL and F using stepwise covariate modeling (SCM) implemented in Perl-speaks-NONMEM (PsN, version 5.3.1). Forward selection [criteria: a decrease in the OFV of at least 3.84 (*P* < 0.05) with one degree of freedom (DF)] and backward elimination [criteria: an OFV increase of at least 7.88 (*P* < 0.005) with one DF] of the covariates were evaluated by the likelihood ratio test assuming X^2^ distribution [27].

The following covariates were tested on TAC CL and F: age [> 65 vs ≤ 65 years]; actual body weight and ideal body weight (kg) at baseline as a continuous variable; sex [female vs male], conditioning regimen [reduced intensity conditioning vs myeloablative (full preparative) conditioning]; HLA match; pharmacogenomic variants CYP3A5*3 [*3/*3 vs *1 carriers]; POR*28 [*1/*1 vs *1/*28 vs *28/*28], CYP3A4*1B [*1/*1 vs *1/*1B], *ABCB1 1236C_T* [*C/C* vs *CT* vs *T/T*]; longitudinal concomitant medication use [yes/no] at each TAC concentration measurement (voriconazole, posaconazole, fluconazole, sulfamethoxazole-trimethoprim, corticosteroids, dihydropyridine calcium channel blockers [amlodipine, nifedipine]; and laxatives [tested only on the logit (F)]). Biochemical laboratory tests were treated as longitudinal, time-varying covariates. For each TAC concentration, the laboratory tests collected at the same day of pharmacokinetic assessment were assigned, otherwise, the values collected at the closest time to pharmacokinetic assessment were carried over. Each biochemical test covariate was subsequently categorized into predefined groups. The collected biochemical tests were estimated glomerular function rate (eGFR) calculated by race-free equation from the observed serum creatinine [28] (< 60 vs ≥ 60 mm/min/1.73m^2^), bilirubin [normal vs high (> 1.3 mg/dL) vs low (< 0.2 mg/dL)], hemoglobin [normal vs low (< 13.3 g/dL (male), < 11.7 (female) g/dL)], hematocrit [normal vs low (< 40% (male), < 35% (female))], blood urea nitrogen [normal vs high (> 30 mg/dL) vs low (< 7 mg/dL)] and albumin [normal vs low (< 3.4 g/dL)]. Approximately 99% of the lab values in serum creatinine, blood urea nitrogen, hemoglobin, and hematocrit were recorded at the time of each TAC concentration. CYP3A5 *6 and *7 were not analyzed as no participants carried these variants. *ABCB1 1236C* > *T* (rs1128503) and *2677C* > *A/T* (rs2032582) variants were not included because they were not available on the genotyping array. CYP3A4*22 was not included in covariate analysis because of the small number of *22 carriers. Linear models were used to test categorical covariates on both CL (Eq. 7) and F (Eq. 8). Actual body weight and ideal body weight were tested in separate SCM iterations using the power model.

7 $$\begin{array}{cc}{COV}_{\begin{array}{c}eff\\ \left(cat,i\right)\end{array}}\left\{\begin{array}{c} 1\;at\;reference\; level \\ 1+ {\uptheta}_{\left(cat=1\right)} covariate\; at\; level\; 1, \\ \dots \dots . etc\end{array}\right.& categorical\; covariate\; (COV)\; modeling\; on\; CL\end{array}$$

8 $$\begin{array}{cc}{COV}_{\begin{array}{c}eff\\ \left(cat,i\right)\end{array}}\left\{\begin{array}{c}0\; at\; reference\; level \\ 0+ {\uptheta}_{\left(cat=1\right)} covariate\; at\; level\; 1, \\ \dots \dots . etc\end{array}\right.& categorical\; COV\; modeling\; on \;F\end{array}$$

Before testing individual species in the population pharmacokinetic model, microbiome alpha diversity indices (Shannon and Inverse-Simpson) were evaluated for correlation with Empirical Bayes estimates (EBEs) in IIV of CL and Logit(F). The alpha diversity indices of the samples collected before or at first oral trough in steady state (*n* = 28) were not significantly different compared to samples collected after first oral trough in steady state (*n* = 16) [median Shannon index = 2.48 (before/at first oral steady state trough) vs 2.23 (after first oral steady state trough), p = 0.6; median Inverse Simpson index = 8.54 (before/at first oral steady state trough) vs 5.95 (after first oral steady state trough), p = 0.5, Wilcoxon test]. Therefore, alpha indices from the samples collected before or at first oral TAC trough in steady state and those collected after were combined and treated as single covariate in the covariate testing.

### Microbiome Processing and Selection of Top Predictive Species with Machine Learning

Bacterial relative abundances were evaluated in each stool sample and species occurring in < 30% of samples or with a mean relative abundance of < 0.1% were removed from further analysis. Relative abundances of the remaining taxa (32 bacterial species [species names are presented in supplement Fig. 1] out of 897 identified bacterial species) were transformed using centered log-ratio (CLR) after replacing zeros with a pseudo number equal to half of the smallest relative abundance in the data [29].

Least Absolute Shrinkage and Selection Operator (LASSO), a machine learning regression approach, was then used to identify the top predictive bacterial species associated with TAC CL and F from the 32 species. Separate LASSO regressions were performed on the EBEs of CL IIV and F IIV obtained from the SCM model that included other covariates with CLR-transformed relative abundances as predictors. The LASSO lambda hyperparameter was optimized using leave-one-out cross-validation. The top predictive microbiome species identified for CL IIV and F IIV were then introduced individually as CLR-transformed continuous covariates in the SCM model using the full fixed effect modeling (FFEM) [30] approach to build the final model.

The microbiome effects were modeled while centered to the median CLR-transformed relative abundance using exponential models for the CL (Eq. 9) and linear models for the logit(F) (Eq. 10).

9 $$\begin{array}{cc}{COV}_{eff}={e}^{{\theta}_{COV}*(COV- {COV}_{median})}& \left[microbiome\; effect\; on\; CL\right]\end{array}$$

10 $$\begin{array}{cc}{COV}_{eff}={\theta}_{COV}*\left(COV-{COV}_{median}\right)& [microbiome\; effect\; on\; F]\end{array}$$

Multiplicative covariate effects were used to model the influence of covariates on CL, whereas additive covariate effects were used for covariates on logit(F).

### Model Evaluation and Diagnostics

The 95% confidence intervals (CIs) from sampling-importance-resampling (SIR) were used to evaluate the precision of the final model parameter estimates [31, 32]. SIR diagnostic plots are reported in supplementary file 2. The model fitting was evaluated by standard goodness-of-fit plots using pmplot package (v 0.5.1) and prediction-corrected visual predictive checks (pcVPC) were conducted using 1000 simulation replicates on PsN [33], and were plotted using tidyvpc package (v 1.5.2).

### Simulations of Covariate Effects on Oral TAC Troughs

To understand and interpret the potential clinical magnitude of the microbiome effect on oral TAC troughs, simulations were conducted using a *ceteris paribus* approach [34]. Simulations were based on the final model using the mrgsolve (v1.6.1.9) and pmforest packages (v0.2.1), with parameter uncertainties coming from the SIR output. The effects of the CLR-transformed bacterial relative abundances on CL and F were first simulated and presented as forest plots. The combined effects of these parameters were then simulated on oral C_t,ss_. The reference for the simulations was oral TAC administered at a daily dose of 3 mg, without co-administration of interacting concomitant medications, CLR-transformed microbiome relative abundances fixed at the median values from the population, and CYP3A5*3/*3 diplotype. CLR-transformed relative abundance values at 95th, 75th, 25th and 5th percentiles were selected for simulation to illustrate the potential differences in effect that species relative abundance may have on oral troughs, CL and F. The impact of each unit increase in the identified bacterial species relative abundance on oral TAC troughs was simulated.

### Statistical Analysis

**Fig. 1:**
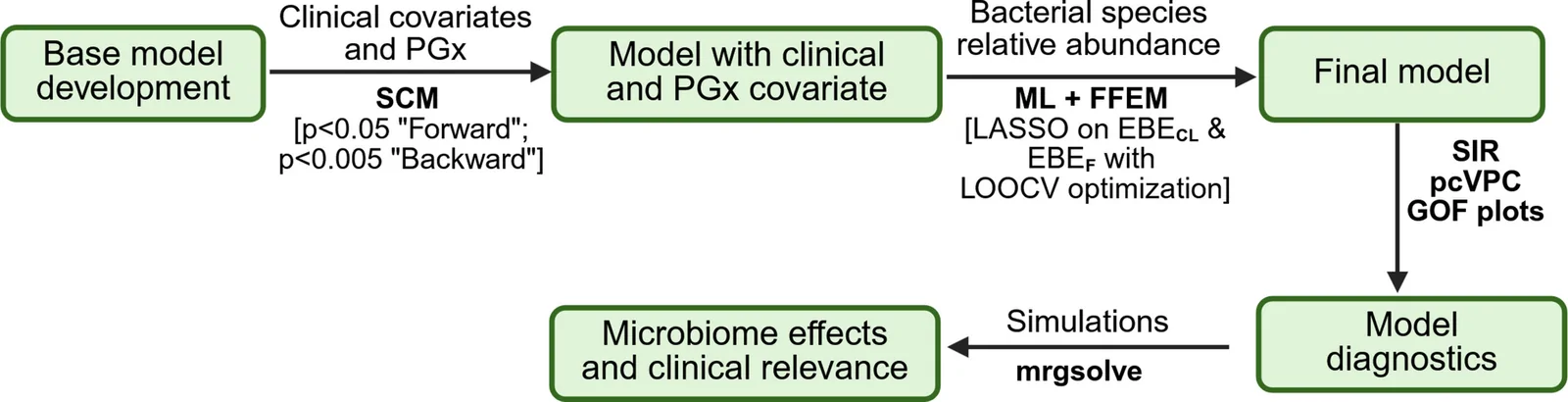
**Schematic diagram of population pharmacometrics modeling.** Population pharmacokinetic modeling and simultaneous estimation of CL and F from IV and oral TAC steady state concentrations was conducted using NONMEM (v7.5). After the base model was developed using the continuous IV infusion model, the clinical factors and pharmacogenomic covariates were assessed using SCM implemented in PsN (v5.3.1). The EBEs of the IIV on CL and F were tested individually for association with microbiome relative abundance using LASSO regression which was optimized using LOOCV. The top predictive species selected by LASSO regression were introduced to the SCM model to build the final model using full fixed effect modeling approach. Final model diagnostics were conducted using SIR for estimate uncertainties, pcVPC (1000 simulations) and GOF plots. The microbiome effects were simulated using mrgsolve and represented as forest plots to assess their significance and clinical relevance. *PGx* = pharmacogenomics; *SCM* = stepwise covariate modeling; *ML* = machine learning; *FFEM* = full fixed effects modeling; *LASSO* = Least Absolute Shrinkage and Selection Operator regression; IIV = inter-individual variability; *LOOCV* = leave-one-out cross validation; *SIR* = sampling-importance resampling; *EBEs* = empirical bayes estimates; *pcVPC* = prediction corrected visual predictive checks; *GOF* = goodness-of-fit.

Descriptive statistics were summarized as median and range for continuous data and frequency and percentages for categorical data. All statistical analyses, plots and simulations were conducted using R (version 4.4.0, Vienna, Austria) on RStudio software (version 2024.04.2, Massachusetts, US). A schematic diagram showing the pharmacometrics modeling approach is presented in Fig. 1.

## Results

### Patient Characteristics

Forty-four HCT recipients met inclusion and exclusion criteria and had sufficient pretransplant DNA and stool specimens available. Seventy-five % (*n* = 33) of the participants were ≤ 65 years old. All participants, except two, self-reported as Caucasian. Acute myeloid leukemia and myelodysplastic syndromes were the most common diagnosis (34.1% and 29.5%, respectively). Reduced intensity conditioning regimen was used in 29 participants (63%), while 15 (34.1) had myeloablative (full preparation) regimen. CYP3A5 *3/*3 and CYP3A4 *1/*1 were the most common genotypes in the participants at 77.3% and 81.8%, respectively. Only three participants carried a CYP3A4*22 variant (6.8%). Almost half-of the participants had POR*1/*28 (54.65%) and *ABCB1* (*1236C* > *T*) *C/T* (54.65%) diplotypes, while only 6 participants (13.64%) had CYP3A4*1/*1B. The demographics and baseline characteristics of the study participants are summarized in Table I.

**Table I:** Demographics and Baseline Characteristics

Variable	Summary statistics (*n* total = 44)
**Categorical variables, *n* (%)**	
Age	
> 65 years	11 (25)
≤ 65 years	33 (75)
Sex	
Female	13 (29.55)
Male	31 (70.45)
Self-reported ancestry	
Caucasians	42 (95.45)
Native American	2 (4.55)
Diagnosis prior to HCT	
Acute Myeloid Leukemia	15 (34.1)
Myelodysplastic Syndromes	13 (29.5)
Acute Lymphoblastic Leukemia	7 (15.9)
Chronic Myeloid Leukemia	2 (4.5)
Chronic Myelomonocytic Leukemia	2 (4.5)
Other	5 (11.4)
Conditioning regimen	
Reduced Intensity Conditioning	29 (63)
Myeloablative (Full Preparative) Conditioning	15 (34.09)
Non-myeloablative^a^	1 (2.27)
CYP3A5*3 [rs776746]	
*3/*3	34 (77.27)
*1/*3	8 (18.18)
*1/*1	2 (4.55)
CYP3A4*1B [rs2740574]	
*1/*1	36 (81.82)
*1/*1B	6 (13.64)
N/A	2 (4.55)
CYP3A4*22 [rs35599367]	
*1/*1	41 (93.18)
*1/*22	3 (6.82)
POR*28 [rs1057868]	
*1/*1	18 (40.91)
*1/*28	24 (54.55)
*28/*28	2 (4.55)
*ABCB1*(*1236C_T*) [rs1128503]	
*C/C*	14 (31.82)
*C/T*	24 (54.55)
*T/T*	6 (13.64)
HLA matching	
8/8	32 (72.73)
6/6	2 (4.55)
4/8	7 (15.91
5/8	1 (2.27)
5/6	1 (2.27)
N/A	1 (2.27)
Number of TAC concentrations (*n* = 658)	
Continuous IV infusion steady state concentration (C_ss_)	222
Oral steady state trough (C_t,ss_)	436
**Continuous variables, median [range]**	
Actual body weight at baseline (kg)	86.7 [46.5, 160.7]
Ideal body weight at baseline (kg)	69.6 [43.2, 80]
Time to first oral TAC dose (days)	16 [7,33]
Time post-HCT to stool sample (days)	14 [11, 31]
Time post-HCT to the first oral steady state trough (days)	19 [10, 35]
Time from stool sample to first oral TAC dose (days)	−2 [−13, 7]
Time from stool sample to first oral steady state trough (days)	1 [−8, 9]
TAC continuous IV infusion rate (mcg/hr)	86 [30, 138]
TAC oral daily dose (mg)	3 [0.5, 15]
TAC continuous IV infusion steady state concentration (ng/ml)	10.85 [3.6, 20.8]
TAC oral steady state trough (ng/ml)	8.9 [1.5, 27.5]
Number of TAC concentration measurements per patient	
Continuous IV infusion Css	5 [2, 11]
Oral steady state troughs	11 [3, 17]

^a^combined with reduced intensity conditioning during the analysis

*HLA* = human leukocyte antigens; *HCT* = hematopoietic stem cell transplant; *TAC* = tacrolimus; *IV* = intravenous; *Css* = steady state concentration; *N/A* = not available “missing”

The median TAC IV continuous infusion dose was 86 mcg/hr representing a daily infusion of 2.064 mg/day, while the median oral daily dose was 3 mg/day divided in two daily doses. A total number of 658 steady state TAC concentrations were analyzed and were within the early period post-HCT representing time on IV infusion and the first 60 days after first oral TAC trough in steady state. A median number of 5 steady state concentrations while on continuous IV infusion and 11 steady state troughs on oral dosing were evaluated per participant. The first oral TAC dose was administered at a median of 16 days post HCT while the median time to first oral steady state trough was recorded at a median of 19 days post-HCT. The stool samples were collected at a median time of 14 days post-HCT and 1 day from the time of first oral steady state trough concentration.

A eGFR of ≥ 60 ml/min/1.73 m^2^ was observed at the majority of the TAC concentrations (*n* = 563, 85.6%). Almost all of the bilirubin measurements were within the normal range (*n* = 627, 95.29%) and nearly all hemoglobin and hematocrit measurements were low (*n* = 644 (97.9%) and *n* = 647 (98.18%), respectively). More than half of the measured albumin levels were low (*n* = 367, 55.8%). Voriconazole (*n* = 296, 45%) and a dihydropyridine calcium channel blocker (*n* = 276, 42%) were commonly used in our participants. The longitudinal biochemical tests and concomitant medications used at the time of the TAC concentration measurements are reported in Table II.

**Table II:** Longitudinal Biochemical Tests and Concomitant Medication Use at Time of Tacrolimus Concentration Measurements

Longitudinal covariates	Number of observations (*n* total = 658), n (%)
eGFR*	
≥ 60 ml/min/1.73m^2^	563 (85.56)
< 60 ml/min/1.73m^2^	95 (14.44)
Bilirubin	
Normal (0.2–1.3 mg/dL)	627 (95.29)
High (> 1.3 mg/dL)	23 (3.50)
Low (< 0.2 mg/dL)	8 (1.22)
Hemoglobin	
Low (< 13.3(M), < 11.7(F) g/dL)	644 (97.87)
Normal	14 (2.13)
Hematocrit	
Low (< 40 (M), < 35(F) %)	647 (98.18)
Normal	10 (1.52)
N/A	1 (0.3)
Blood urea nitrogen	
Normal (7–30 mg/dL)	584 (89.02)
High (> 30 mg/dL)	56 (8.54)
Low (< 7 mg/dL)	16 (2.44)
Albumin	
Normal (> 3.4 g/dL)	291 (44.22)
Low (< 3.4 g/dL)	367 (55.78)
No. of concentrations with voriconazole use	296 (44.98)
No. of concentrations with posaconazole use	110 (16.72)
No. of concentrations with fluconazole use	133 (20.21)
No. of concentrations with sulfamethoxazole/trimethoprim use	162 (24.62)
No. of concentrations with corticosteroids use	157 (23.86)
No. of concentrations with dihydropyridine CCB (amlodipine or nifedipine) use	276 (41.95)
No. of concentrations with laxatives use^a^	66 (10.03)

^a^Laxatives: Sennosides/docusate sodium, Polyethylene glycol, Sennosides, Docusate sodium, Psyllium

^*^calculated using 2021 CKD-EPI race free equation [28]

*CCB* = calcium channel blockers; *N/A* = not available “missing”

### Microbiome Characteristics

**Fig. 2:**
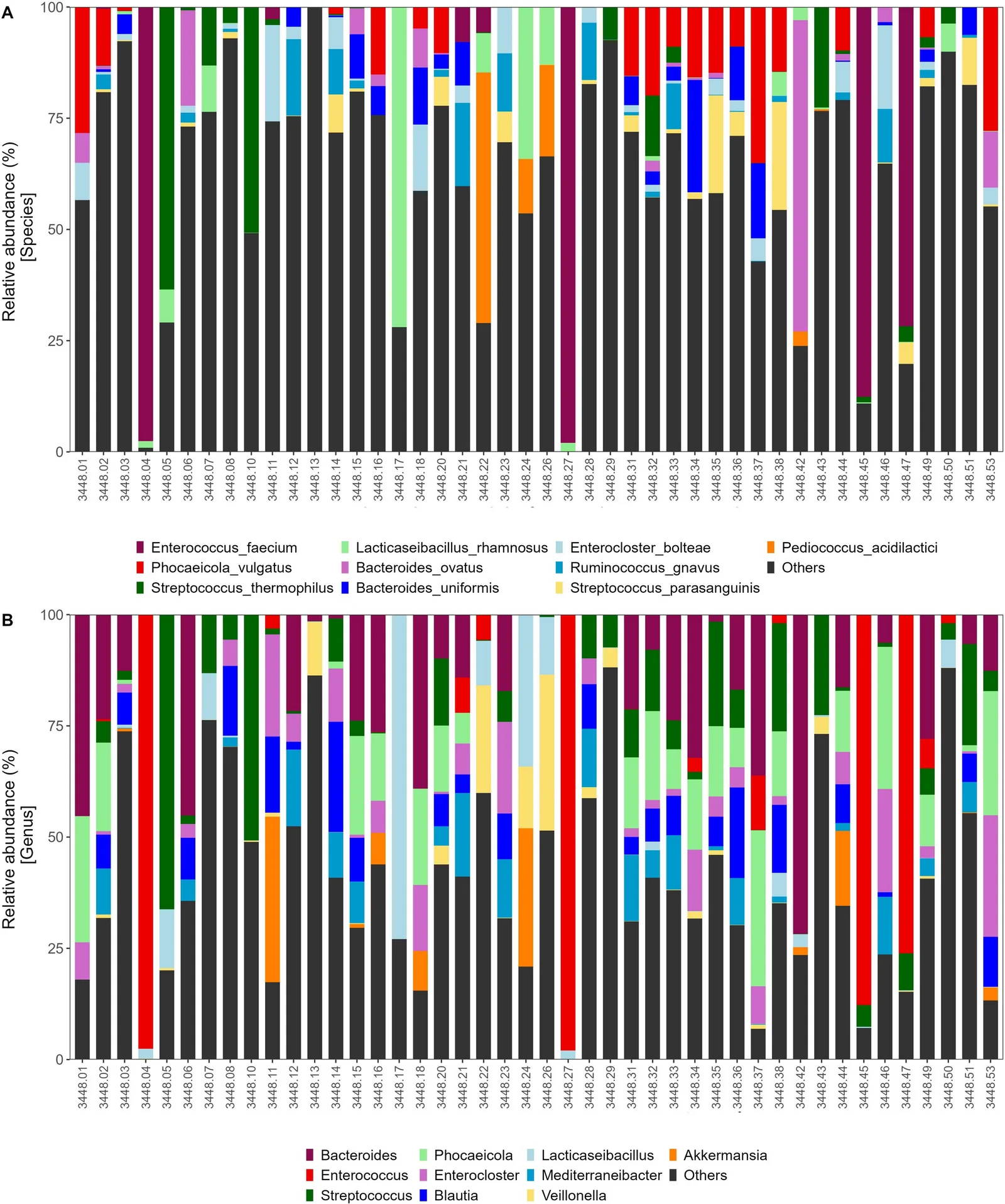
**Bar plot for the top 10 species and genre identified in the stool of study participants at time of first steady state oral TAC trough.** The stacked plots represent the bacterial relative abundance before processing (i.e. before removing less prevalent taxa) for the identified (**A**) species level (*n* = 897) and (**B**) genus level (*n* = 449). The abundance of bacterial taxa is highly variable among the study participants and in some participants the microbiome is dominated by one or two taxa only.

Metagenomic shotgun sequencing identified 897 bacterial species in the 44 stool samples. The microbiome profiles showed high heterogeneity across the study participants as represented in the bar plots of the species and genus relative abundances, Fig. 2. *Enterococcus faecium* had the highest relative abundance and was driven by 4 participants where it was the predominant species in their stool with a relative abundance of > 75%. Substantial dominance of *Enterococcus* or *Streptococcus* genera was observed in some HCT recipients in our sample, Fig. 2 (Plot B). After removing infrequent taxa occurring in < 30% of stool samples or those with a mean and very small relative abundance of < 0.1%, 32 species remained for analyses. To avoid the potential multi-collinearity associated among the 32 species, the CLR-transformed relative abundances were tested for correlation (Supplement Fig. 1). None of the analyzed species were highly correlated (rho > 0.8). Among our samples, no statistically significant differences in the abundance of species were observed between samples collected before or at time of first oral steady state TAC trough (*n* = 28 samples) and those collected after first oral TAC trough in steady state (*n* = 16 samples) (Supplement Fig. 2).

In a subgroup analysis (*n* = 35), we found that the alpha diversity indices increased from day 14 to day 28 (Supplement Fig. 3, plot A). Shannon index increased from a median of 2.06 to 2.61 (*p* = 0.004, Wilcoxon signed-rank test) while inverse Simpson increased from a median of 5.41 to 8.16 between stool samples on day 14 to day 28, respectively (*p* = 0.047, Wilcoxon signed-rank test). Despite the increase in alpha diversity, none of the identified species were differentially abundant between the stool samples obtained at day 28 compared to samples obtained at day 14 (Supplement Fig. 3, plot B).

### Final Population Pharmacokinetic Model, Significant Clinical and Microbiome Covariates

The population TAC CL was estimated to be 6.91 L/hr and the population oral F was 64.4%. *CYP3A5* genotype, voriconazole and eGFR were important determinants of TAC CL. Compared to CYP3A5*3/*3, having one or more CYP3A5*1 allele is associated with 57.5% increase in TAC CL, while voriconazole use or having eGFR < 60 ml/min/1.73 m^2^ were associated with decreased CL (−20.3% and −16.9%, respectively). Corticosteroid use was associated with a decrease of 0.447 in logit(F) and laxative use was significantly associated with an increase of 0.527 in logit(F). Estimates are shown in Table III.

**Table III:** Final Tacrolimus Model Estimates

Parameter^a^			Estimate	SIR, median [95%CI]	RSE %	Shrinkage %
Fixed effects	Structural model	Clearance (CL) [L/hr]	6.91	6.961 [6.4,7.51]	4.25	-
		Oral bioavailability (F)^#^	0.644	0.647 [0.565,0.716]	28.73^$^	-
	Clinical and PGx covariates	CL ~ CYP3A5 *1 carrier	0.575	0.578 [0.357,0.818]	20.63	-
		CL ~ Voriconazole use	−0.203	−0.202 [−0.279,−0.127]	19.43	-
		CL ~ eGFR < 60 ml/min/1.73 m^2^	−0.169	−0.172 [−0.25,−0.089]	25.35	-
		logit(F) ~ Corticosteroid use	−0.447	−0.439 [−0.672,−0.209]	27.1	-
		logit(F) ~ Laxative use	0.527	0.543 [0.166,0.969]	40	-
	Microbiome covariates^a^	CL ~ *Bacteroides uniformis*	0.0203	0.02 [0.004,0.035]	39.1	-
		CL ~ *Bacteroides ovatus*	0.0113	0.011 [−0.002,0.025]	61.71	-
		CL ~ *Limosilactobacillus fermentum*	−0.0345	−0.035 [−0.05,−0.02]	20.86	-
		logit(F) ~ *Ruthenibacterium lactatiformans*	0.12	0.124 [0.046,0.191]	29.96	-
		logit(F) ~ *Veillonella atypica*	0.0505	0.052 [−0.012,0.111]	58.7	-
		logit(F) ~ *Sellimonas intestinalis*	0.0508	0.052 [−0.009,0.109]	59.4	-
		logit(F) ~ *Enterococcus faecium*	−0.0605	−0.06 [−0.104,−0.018]	35.68	-
		logit(F) ~ *Bacteroides uniformis*	−0.0678	−0.072 [−0.124,−0.021]	37.78	-
		logit(F) ~ *Bacteroides ovatus*	−0.0591	−0.058 [−0.102,−0.014]	36.86	-
		logit(F) ~ *Limosilactobacillus fermentum*	0.0552	0.058 [0.005,0.106]	45.38	-
		logit(F) ~ *Eggerthella lenta*	0.139	0.14 [0.073,0.22]	26.21	-
		logit(F) ~ *Blautia caecimuris*	−0.115	−0.113 [−0.182,−0.05]	28.45	-
		logit(F) ~ *Streptococcus salivarius*	−0.0724	−0.071 [−0.144,−0.003]	47.49	-
Random effects	II variance	IIV-CL	0.0261 [CV% = 16.3]	0.027 [0.015,0.049]	32.85	18.6
		IIV-logit(F)	0.208 [SD = 0.101]	0.257 [0.131,0.389]	49.08	23.2
	RU variance	Proportional error	0.122 [CV% = 35]	0.122 [0.108,0.139]	6.51	3.23

^a^ For CL, the microbiome effects (e^θ^) represent the relative change in CL associated with each 1-unit increase in centered CLR-transformed relative abundance from the median, whereas for logit(F), the microbiome effects (θ) represent the absolute change in logit(F) associated with each 1-unit increase in centered CLR-transformed relative abundance from the median

^$^The RSE is reported for the TVF1 on logit scale and before inverse-transformation

^#^F = exp(logit(F))/(exp(logit(F)) + 1), F was modeled as logit-normal distribution to ensure values between 0 and 1

$$CV\%\;of\;\log-normal\;omega=\sqrt{e^{estimate}-1}\ast100$$

$$CV\%\;of\;sigma=\sqrt{estimate}\ast100$$

SD of logit-normal omega was calculated using logitnorm package implemented in pmparms package in R

*PGx* = pharmacogenomics; *II* = inter-individual, *RU* = residual unexplained, *IIV* = inter-individual variability; *CL* = clearance; *F* = oral bioavailability; *SD* = standard deviation; *CV* = coefficient of variation; *RSE* = relative standard error; *SIR* = sampling importance resampling

The IV and oral TAC concentrations were modeled simultaneously and therefore the differential effect of *CYP3A5* on IV infusion vs oral TAC exposure was not determined. To assess for potential differences, we analyzed the distribution of the log-transformed dose-adjusted TAC concentrations and TAC concentrations, versus *CYP3A5* diplotypes (*1/*1 vs *1/*3 vs *3/*3) by route of administration, Supplement Fig. 4. There was a similar magnitude of effect on TAC concentrations in each diplotype group on the IV continuous infusion and oral routes of administration.

Using machine learning (LASSO regression), three bacterial species were possibly predictive of TAC CL (Supplement Fig. 5, plot A) and 10 bacterial species (*Ruthenibacterium lactatiformans, Veillonella atypica, Sellimonas intestinalis, Enterococcus faecium, Bacteroides uniformis, Bacteroides ovatus, Limosilactobacillus fermentum, Eggerthella lenta, Blautia caecimuris,* and *Streptococcus salivarius*) for F (Supplement Fig. 5, plot B). Three of those species (*Bacteroides uniformis, Bacteroides ovatus* and *Limosilactobacillus fermentum)* were identified to be predictive of both CL and F. These species did not show any multicollinearity when tested through variance inflation factor analysis (Supplement Fig. 6).

The identified species were then tested in the final population pharmacokinetic model. The final model with clinical covariates and microbiome effect sizes are presented in Table III. Although significant in the LASSO model, the 95% CI of the effects of *Bacteroides ovatus* on CL, and *Veillonella atypica* and *Sellimonas intestinalis* on Logit(F) include the null value (0). These species were left in the model to maintain model stability and their simulated effects are not shown in the results. In the final model, two bacterial species, *Bacteroides uniformis* and *Limosilactobacillus fermentum*, were important toward CL; however, the magnitude of effect was modest. Specifically, each unit increase in the CLR-transformed relative abundance from the median was associated with change in CL with an increase of 2.05% [% change = (e^0.0203^ − 1) × 100] for *Bacteroides uniformis* and a 3.39% decrease [% change = (e^−0.0345^ − 1) × 100] for *Limosilactobacillus fermentum*. Eight species (*Ruthenibacterium lactatiformans, Enterococcus faecium, Bacteroides uniformis, Bacteroides ovatus, Limosilactobacillus fermentum, Eggerthella lenta, Blautia caecimuris,* and *Streptococcus salivarius)* were identified towards Logit(F). Among these, *Eggerthella lenta* and *Ruthenibacterium lactatiformans* demonstrated the largest positive effects with increases of 0.139 and 0.12, respectively, while *Blautia caecimuris* had the largest negative effects corresponding to a decrease of 0.115 in Logit(F). Weak and non-significant correlations were observed between alpha diversity and EBEs in CL and logit(F), Supplement Fig. 7; therefore, alpha diversity indices were not included in the population pharmacokinetic model.

Including the microbiome in the final model resulted in a decrease in CV% of the CL IIV from 25.8% (a model with only clinical factors, concomitant medications and pharmacogenomics) to 16.3%, and a decrease in the estimated logit(F) IIV variance from 0.885 to 0.208.

### Model Diagnostics

**Fig. 3:**
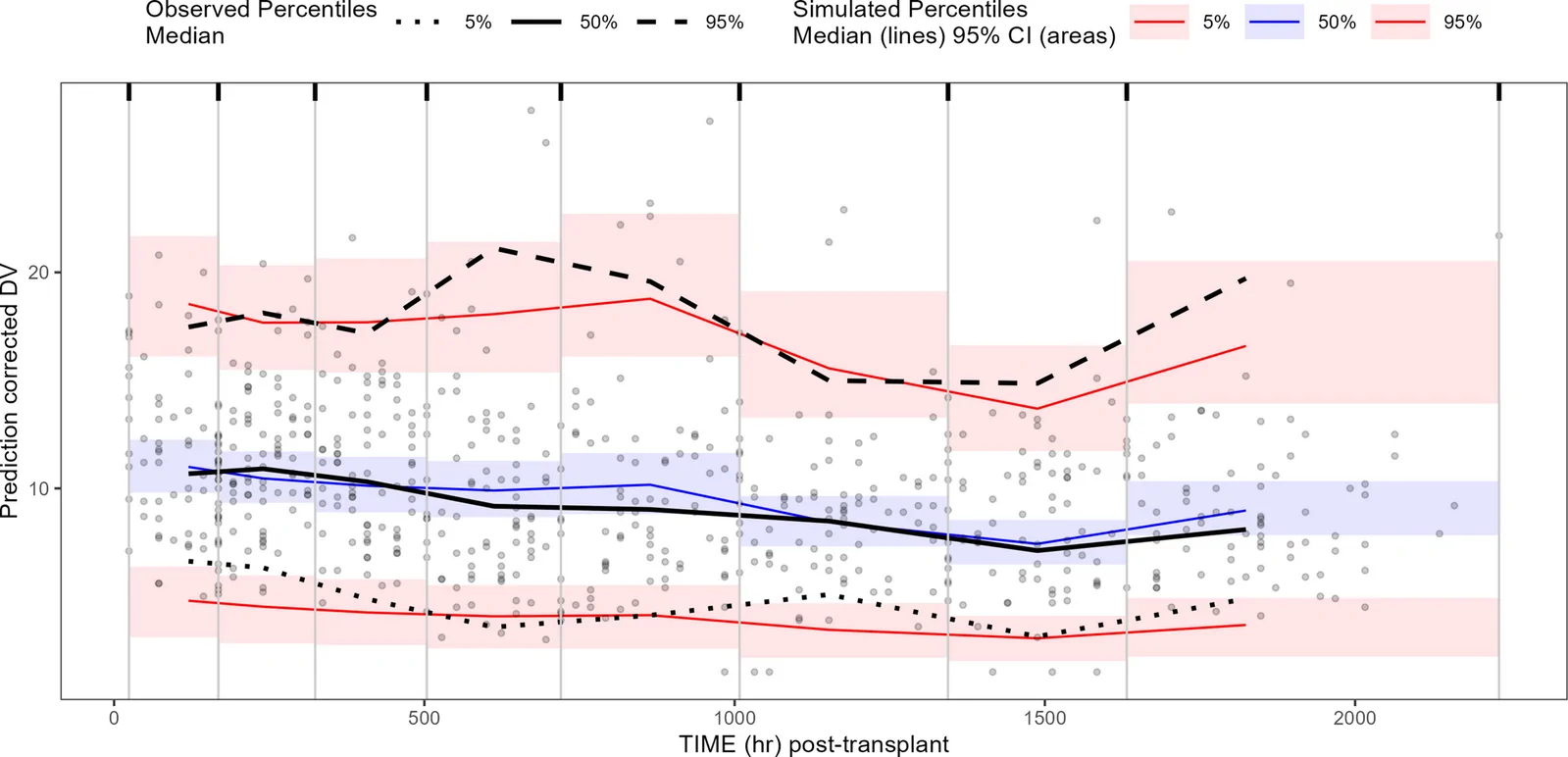
**Prediction-corrected visual predicted checks after 1000 simulations.** *DV* = dependent variable; *TAC* concentrations; *CI* = confidence intervals.

The final model had an acceptable goodness of fit as shown in the diagnostic scatterplots, Supplement Fig. 8. The final model estimates were consistent with the SIR median estimates and contained within the 95% CI supporting model stability. The prediction-corrected visual predictive check showed an adequate and acceptable prediction performance, Fig. 3.

### Simulation of the Microbiome Effects on Oral TAC Troughs

**Fig. 4:**
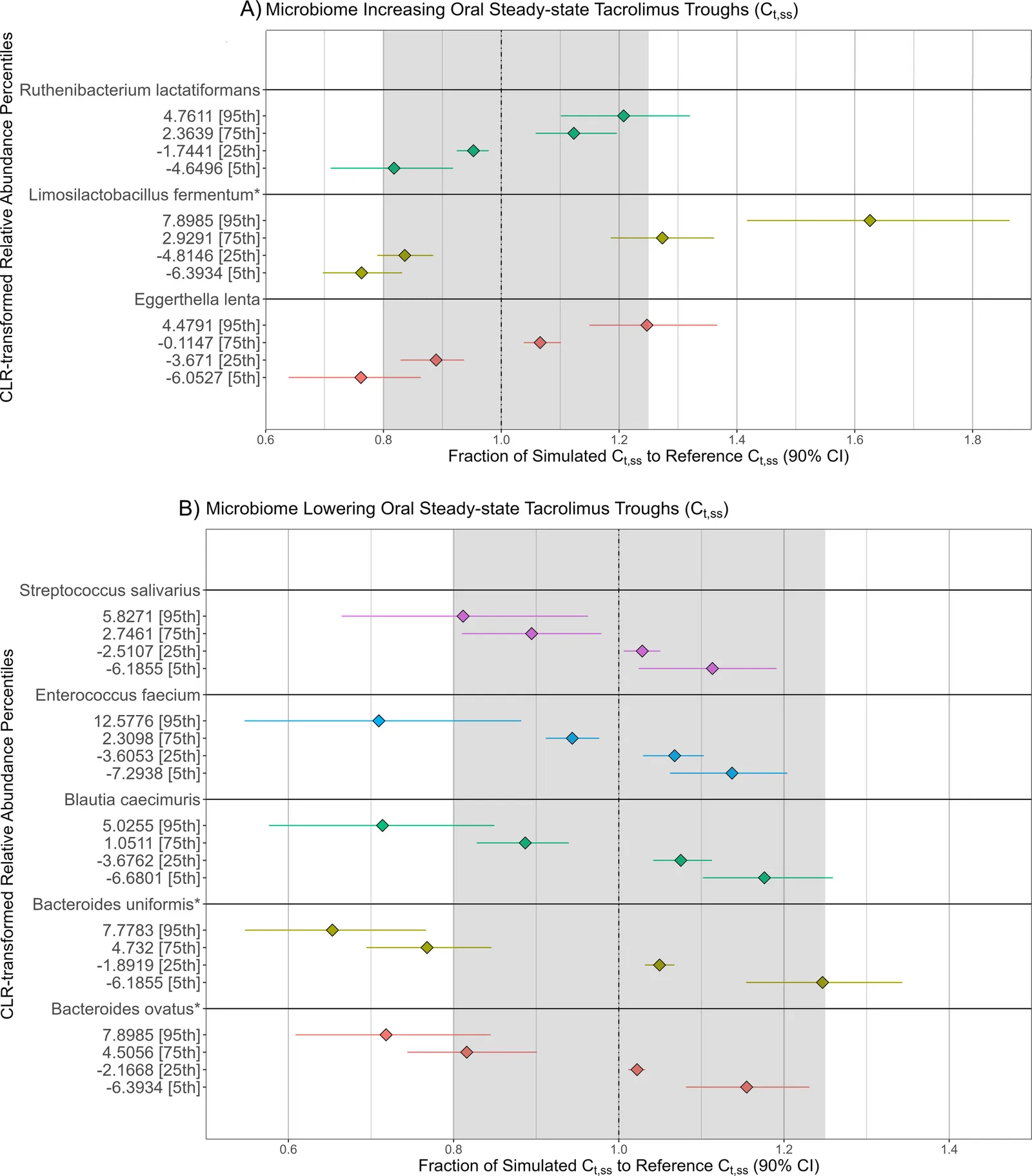
**Simulation forest plot of the effect of the relative abundance of stool microbiome species on oral steady state tacrolimus trough concentrations. **This forest plot shows the effect of varying microbiome relative abundances on the oral TAC trough at steady state with one microbiome altered at a time while fixing the other covariates at their median value. The x-axis is the fraction of simulated oral TAC trough relative to the oral trough at reference values and their 90% CI (reference values are oral daily dose = 3 mg/day, median centered-log ratio (CLR)-transformed relative abundance, CYP3A5*3/*3, without concomitant medication use). The gray shaded area represents a 80–125% variation from the reference oral TAC trough. The vertical dashed line represents the null value at the median CLR-transformed relative abundance. The y-axis is the CLR-transformed relative abundance values ranked at 5th, 25th, 75th and 95th percentiles. If the fraction and its entire 90% CI is outside of the gray shaded area, the simulated effect of the microbiome covariate value was considered clinically relevant. If the 90% CI crosses the boundary between clinically relevant and irrelevant effects, the covariate clinically relevancy is uncertain.

To identify if any bacterial abundances had the potential to result in a clinically relevant effect (defined as 20% or more change) on TAC steady state oral trough concentrations through bacterial-mediated changes in CL and F, simulations were conducted. An increasing relative abundance of 3 bacterial species, *Ruthenibacterium lactatiformans*, *Limosilactobacillus fermentum* and *Eggerthella lenta,* resulted in statistically significant increasing oral TAC troughs, Fig. 4 (panel A). Overall the magnitude of increase by each individual bacteria were modest (< 20% change in trough) except at the highest stool relative abundance (the 95th percentile) of *Limosilactobacillus fermentum* where a clinically relevant increase in TAC troughs of 63% was observed compared to the reference value at the median relative abundance. An increasing relative abundance of 5 bacterial species, *Bacteroides uniformis, Bacteroides ovatus, Enterococcus faecium, Blautia caecimuris and Streptococcus salivarius,* showed a statistically significant association with decreasing simulated TAC troughs at all CLR-transformed relative abundance percentiles, Fig. 4 (panel B). The clinical relevance of these species was generally modest (< 20% change in trough), except at the 95th percentile of abundance of *Bacteroides uniformis, Bacteroides ovatus, Enterococcus faecium* and *Blautia caecimuris*, where a median decrease of 27 to 35% in simulated TAC trough concentrations was observed relative to the reference. The simulations also showed similar association trends with a modest clinical relevance on the change in CL and F at different CLR-transformed relative abundance percentiles, Supplement Figs. 9 and 10.

**Fig. 5:**
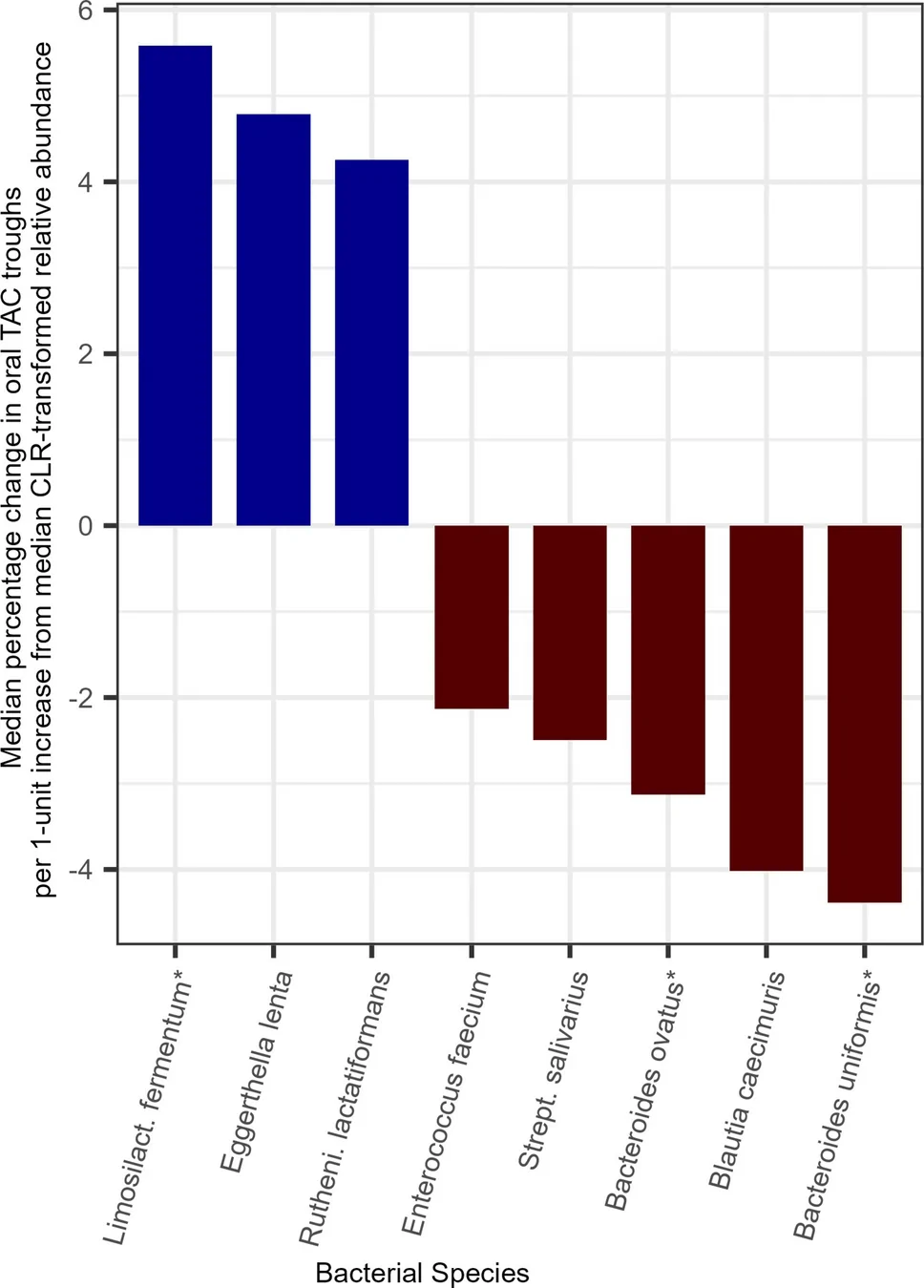
**Simulation of the percentage increase in oral steady state tacrolimus trough concentrations with each one unit increase in bacterial CLR-transformed relative abundance.** Bacterial species with * means they impact both TAC CL and F while the rest impact only TAC F.

To provide an easier interpretation, we also simulated the impact of each unit increase in CLR-transformed relative abundance on oral TAC troughs, Fig. 5. A median percentage increase of ~ 4 to 5.5% in oral TAC troughs were observed with each unit increase in CLR-transformed relative abundance of 3 species with positive associations (*Limosilactobacillus fermentum*, *Eggerthella lenta* and *Ruthenibacterium lactatiformans)*. An ~ 2 to 4.5% decrease in oral TAC troughs were observed with each unit increase in CLR-transformed relative abundance of the 5 species with negative associations. *Bacteroides uniformis* and *Blautia caecimuris* showed the highest median decrease (~ 4 to 4.5%) on oral TAC troughs with each unit increase in CLR-transformed relative abundance.

## Discussion

Tacrolimus is known for its high interpatient pharmacokinetic variability which imposes challenges for dosing. Understanding sources of variability may improve dose selection leading to fewer toxicities and improved efficacy. In transplantation, both clinical factors and pharmacogenomic variables have been identified as contributors to the observed variability in TAC pharmacokinetics; however, a large portion of this variability remains unexplained. In solid organ transplantation, gut microbiome is emerging as a new source of TAC inter-individual variability. Given the profound microbiome disruption following HCT, TAC pharmacokinetics may be particularly susceptible to microbiome-mediated effects, though this area remains unexplored. Integrating clinical factors, pharmacogenomics and microbiome could lead to a better understanding of TAC pharmacokinetics and improve immunosuppression after allogeneic HCT. The current study aimed to identify, for the first time, the effect of the gut microbiome combined with other clinical factors and pharmacogenomics on TAC CL and F in the early post-HCT period.

Only a limited number of studies have evaluated the TAC CL using population pharmacokinetics in adult HCT patients. In our study, we found that TAC CL was 6.9 L/hr which is consistent with the literature and within the reported ranges of 5.22 L/hr [26], 7.94 L/hr [35] and 12.1 L/hr [36]. Our estimated TAC bioavailability (64.4%) was moderate and within the reported ranges. In solid organ transplantation, TAC bioavailability is highly variable, ranging from 5–93% with a mean of 25% [37, 38]. In HCT, TAC bioavailability is inconsistent between studies. Some studies reported low TAC bioavailability of 16% [39], 19% [40] and 28% [26], while others reported higher bioavailability of up to 49% [36, 41]. Many reasons could explain the variability in TAC bioavailability across studies including food intake, concomitant medications and diarrhea. Some data suggest that diarrhea, which commonly occurs post HCT, reduces CYP3A and PGP activity resulting in reduced first-pass effects and increased bioavailability [42]. Similarly, mucositis may lead to a disrupted intestinal barrier resulting in increased permeability and a leaky gut, which could impact TAC absorption and bioavailability [43]. We found in this analysis that the gut microbiome may also explain a higher bioavailability.

Microbiome dysbiosis is common after HCT due to factors such as radiation, chemotherapy and high antibiotic burden. Some studies reported that microbiome dysbiosis can persist for up to 100 days post HCT [44, 45]. Small improvement of the microbiome post HCT was observed in our study as shown in the increase in alpha diversity in the subgroup analysis between stool samples obtained at day 14 and day 28 (Supplement Fig. 3, plot A). Similar improvement in alpha diversity was reported previously post HCT with the lowest value observed around day 14 post HCT [46]. While changes in community structure over time may influence diversity measures such as alpha diversity, they do not necessarily imply corresponding changes in the relative abundance of individual species. We found that the relative abundances of the species identified in the stool were not different across the study period in our subgroup analysis (Supplement Fig. 3, plot B) nor between samples collected before or at first oral TAC steady state trough (*n* = 28) and samples collected after first oral TAC steady state trough (Supplement Fig. 2). Alpha diversity is not directly informative for our research question because the objective of our analysis was to evaluate associations at the level of individual species relative abundance rather than global diversity metrics. Despite this, we tested the alpha diversity as a covariate, and it was not correlated with the IIV in TAC CL and F and therefore, it was not included in the population pharmacokinetic modeling (Supplement Fig. 7). Because there was no differentially abundant species between the stool samples, we assumed that identified species relative abundance in the in the stool samples in our study did not significantly change during early post HCT time. In our study, *Bacteroides* was the predominant genus (Fig. 2). Consistent with our findings, *Bacteroides* was also found to be the most prevalent genus in a study of 20 adult HCT recipients during a similar time period [47]. *Enterococcus faecium* dominated the microbiome profile in 4 HCT recipients in our study. *Enterococcus faecium* domination was previously reported after allogeneic HCT [48, 49]. Similar dominance of the *Streptococcus* genus has been documented previously in HCT recipients, which was also consistent with observations from the current study [12].

Evidence for TAC-microbiome interactions is emerging in solid organ transplantation [11]. In kidney transplant recipients, a higher relative abundance of stool *Faecalibacterium prausnitzii* was associated with higher TAC doses requirements suggesting greater metabolism [14]. While *in vitro*, some gut bacterial species (such as *Blautia hansenii*, *Eubacterium hallii*, *Akkermansia muciniphila*, and *Clostridium sporogenes*) metabolized TAC by more than 50% after 12 h of incubation [17]. In our HCT population, these bacterial species were infrequently observed and/or had a very small relative abundance (i.e., *Faecalibacterium prausnitzii* was present in only 5 participants and had a mean relative abundance of only 2.93 ± 3.98%*)* and were therefore not included in the analysis. In line with our findings, *Faecalibacterium prausnitzii* has been reported to be markedly depleted after HCT, supporting its absence in our patients [50, 51]. Absence of these species could result in a reduced TAC metabolism prior to absorption and hence increase the bioavailable dose leading to an increased bioavailability.

The current study is the first to identify bacterial associations with TAC pharmacokinetics in HCT. Due to the relatively small sample size and lack of validation cohort, our findings on microbiome associations should be considered as hypothesis generating which warrant future in-vitro mechanistic studies and validation with larger cohorts. We identified 8 species potentially important for F and 2 bacterial species for TAC CL. Two of these bacteria, *Limosilactobacillus fermentum* and *Bacteroides uniformis,* affected both CL and F. The effects of these bacteria on CL were small while the effects on F were much more pronounced. These microbial taxa most likely interact with TAC through direct and indirect mechanisms [11]. We speculate that bacterial species associated with decreased TAC bioavailability interact directly with TAC by metabolizing the drug in the gut, thereby reducing the bioavailable dose and overall bioavailability. TAC was found to be directly metabolized by common gut bacteria in solid organ transplant recipients and *in vitro* to a less active bacterial metabolite [15, 17]. Species associated with increased TAC bioavailability were assumed to indirectly influence TAC by inhibiting or reducing PGP activity, mediated by bacterial metabolites. A study by Degraeve *et al.* [20], found that gut microbiome can modulate TAC pharmacokinetics in mice through regulation of PGP via the ABCB1 gene. This modulation was assumed to be mediated through polar bacterial metabolites. We propose that species associated with reduced TAC CL may interact indirectly by modulating CYP3A enzyme expression and/or activity through bacterial metabolites [18, 52].

Most of the species we identified have unknown mechanisms for an interaction with TAC. Among the species with positive association with oral TAC troughs, *Eggerthella lenta* has been reported to inhibit PGP in mice through production of PGP ATPase efflux activity inhibitors [53]. This finding could explain our observation of increased TAC bioavailability associated with *Eggerthella lenta*. TAC is a PGP substrate [38] and inhibition of this transporter would reduce drug efflux and improve bioavailability resulting in increased TAC systemic concentrations. In contrast to our finding, in an *in vitro* study, *Eggerthella lenta* moderately metabolized TAC (~ 44%) when incubated for 12 h [17]. Another species with positive effects, *Limosilactobacillus fermentum* (formally known as *Lactobacillus fermentum*) was reported to improve TAC induced hypertension in mice [54]; however, there are no reports on impact of *Limosilactobacillus fermentum* on TAC exposure and/or impact on transporters or metabolizing enzymes. *Bacteroides uniformis* and *Bacteroides ovatus,* associated with bioavailability and clearance, were reported to metabolize TAC in *in vitro* studies [15, 17]. This is consistent with our findings in which these species are associated with lower oral TAC concentrations. In the current study we found that *Ruthenibacterium lactatiforman* increases bioavailability. *Ruthenibacterium lactatiforman* was recently found to have a moderately negative correlation with *CYP3A4* expression in humanized PXR-CAR-CYP3A4/3A7 mice [52]. Reducing expression of *CYP3A4* could result in reduced first-pass metabolism and hence increased bioavailability. We found that *Streptococcus salivarius* can reduce TAC oral trough concentrations, however in a recent study in liver transplant recipients, *Streptococcus salivarius* was associated with higher TAC concentrations [55]. In our study, we found that high relative abundances of *Enterococcus faecium* species (as in case of microbial dominance) are associated with significantly reduced TAC oral trough concentrations as shown in the simulations in Fig. 4, plot B. Enterococcus faecium domination after allogeneic HCT has been reported before and has been linked to worse outcomes including higher risk of acute GvHD [48, 49]. We propose that this reduction in TAC exposure may lead to insufficient immunosuppression, which could contribute to the increased risks of GvHD and mortality observed in these patients.

The individual effect size of each of the identified species on TAC pharmacokinetics is small and/or modest and may not be considered clinically relevant. However, these bacterial species live in an interdependent ecosystem and, as such, the combined effects may result in larger changes in TAC exposure especially in patients with bacterial dominance. Use of microbiome as biomarker in clinical practice is challenging and hindered by current technology limitations including long turnaround time, extensive and expensive sample processing, and complex bioinformatics and absence of standardization [56]. Stool collection is not yet a part of routine care and self-collection of samples by patients may not be easily achievable due to challenges in sample handling and transportation and the associated high costs. Future advancements in microbiome detection technologies might help identify certain species or a surrogate biomarker which would be easier to implement in clinical practice. Given the emerging evidence of TAC and microbiome interactions, the use of bacterial metabolites would be a cheaper and more feasible alternative to microbiome. Compared with microbiome profiling, bacterial metabolites can be readily detected in plasma with a faster turnaround time using analytical platforms that are already widely available in clinical laboratories.

We identified that pharmacogenomic variants, estimated glomerular filtration rate and voriconazole use and certain stool bacteria species were associated with TAC pharmacokinetics. Genetic variation in *CYP3A5* was the most significant covariate in our study. Carrying one or more CYP3A5*1 alleles (i.e. CYP3A5*1*3 or *1/*1) increased TAC CL by ~ 57.5% compared to CYP3A5*3/*3 (rs776746). Association of *CYP3A5* genetic variation and TAC exposure is known in HCT, and shows higher TAC CL and dose requirements, and lower TAC troughs in CYP3A5*1 carriers compared to CYP3A5*3/*3 [57, 58]. Consistent with our findings, a study by Dunlap *et al.* found that *CYP3A5* intermediate or normal metabolizers (CYP3A5*1/*3 and CYP3A5*1/*1, respectively) had approximately a doubled TAC CL in adult allogeneic HCT patients compared to *CYP3A5* poor metabolizers (CYP3A5*3/3) (0.7 L/hr/kg vs 0.33 L/hr/kg, respectively)[59]. In another study in pediatric HCT recipients, CYP3A5*1 carriers (CYP3A5*1/*3 and CYP3A5*1/*1) had 1.38-fold higher TAC CL compared to *CYP3A5* non-carriers (CYP3A5*3/*3) [40].

We also found that a low eGFR was associated with TAC CL where an eGFR < 60 ml/min/1.73 m^2^ decreased TAC CL by ~ 17%. None of the other published studies tested the association of TAC CL with eGFR although some reported similar associations with serum creatinine. In adult HCT recipients, a serum creatinine ≥ 2 mg/dl was associated with 40% reduction in TAC CL [26]. In pediatric HCT recipients, SCr was inversely associated with TAC CL [39]. TAC is mostly (95%) eliminated through biliary route and ~ 2.4% renally eliminated [60] therefore, the mechanism of this relationship is unclear. However, several mechanisms may explain this association. First, impaired kidney function, reflected by lower eGFR, is associated with accumulation of uremic toxins, which may inhibit CYP3A4 activity and consequently reduce TAC CL [61]. Another possible explanation is reverse causality, whereby higher TAC exposure, resulting from lower TAC CL, increases the risk of TAC-induced nephrotoxicity and subsequent reduction in eGFR.

We found that voriconazole use was associated with a 20% decrease in TAC CL. Tacrolimus is primarily metabolized by CYP3A5 and CYP3A4 [62]. Voriconazole is a moderate to strong CYP3A4 and CYP2C19 inhibitor [63] and the voriconazole interaction with TAC is documented in transplantation [64]. Interestingly, posaconazole which is also a strong CYP3A4 inhibitor [65] did not influence TAC CL in our study. This could be due to the limited use of posaconazole in our population and approximately 32% of these observations associated with posaconazole were in *CYP3A5* expressers (*1/*1 or *1/*3). The inhibition of TAC clearance through CYP3A4 by posaconazole may be less pronounced in *CYP3A5* expressers compared with *CYP3A5* non-expressers (*3/*3), in whom TAC metabolism relies mainly on CYP3A4. Aligning with our assumptions, solid organ transplant recipients who are *CYP3A5* expressors were found to have a degree of resistance to the inhibitory effects of posaconazole on TAC exposure [66].

We found that TAC bioavailability is impacted by use of laxatives and corticosteroids. Laxative use was associated with increased TAC bioavailability, but the mechanism of this interaction is unclear. Some laxatives such as senna/sennosides can inhibit PGP and may reduce TAC efflux and increase bioavailability. In animal models, senna laxatives were also found to inhibit PGP function in the intestine of rats [67]. Similarly, a case report in a liver transplant recipient developed supratherapeutic TAC concentrations during co-administration with *Cassia angustifolia* herbal product (senna containing laxative) [68]. We also identified reduced TAC bioavailability with corticosteroids use. Corticosteroids can induce PGP and CYP3A activity which could result in increased TAC efflux and first pass metabolism in the intestine which would lead to a decreased TAC bioavailability [69]. The inverse interaction between TAC and corticosteroids has been reported previously. Kidney transplant recipients receiving steroids required higher TAC doses [69], and Staatz et al. [70] reported that the corticosteroid effect on TAC pharmacokinetics was better characterized by a decrease in tacrolimus bioavailability rather than an increase in clearance.

The current study comes with limitations. The information on mucositis, gastritis and diarrhea and their severity was not available and not included in the analysis. Future studies should be designed to collect this information as it may improve pharmacokinetic models, especially in HCT setting [43]. Future studies with larger sample sizes are needed to validate our findings. The current study included only a single timepoint for stool microbiome analysis which may not reflect the relative abundance at later times post HCT. Future studies should be designed to include longitudinal microbiome samples. Finally, oral TAC trough concentrations were extracted from electronic medical records and timing of blood draws were not supervised by our study. Receiving medication at different times than prescribed and/or missing doses could potentially impact bioavailability and clearance estimates.

## Conclusion

Genetic variation in *CYP3A5*, voriconazole use and kidney function impacts TAC clearance while corticosteroid and laxative use appear to impact TAC oral bioavailability in adult HCT recipients. We also identified 8 bacterial species associated with TAC clearance and bioavailability including two species (*Limosilactobacillus fermentum* and *Bacteroides uniformis*) affecting both. Future validation studies with larger sample sizes and multiple microbiome samples are needed. Studies like the present study are critical to identify potential bacterial species associated with drug pharmacokinetics from the hundreds of species present within the gut microbiome. Such data can inform targeted bacterial metabolite studies and *in vitro* functional studies, ultimately helping to identify the specific organisms most relevant to TAC disposition. We envision in the future that targeted bacterial metabolite tests will be a practical approach for clinical microbiome monitoring compared to metagenomic shotgun sequencing which have high cost and long turnaround time. 

## Supplementary Information

Below is the link to the electronic supplementary material.

Supplementary file1 (DOCX 1848 KB)

Supplementary file2 (PDF 183 KB)

Supplementary file3 (CSV 113 KB)

## Data Availability

Data will be available upon reasonable request from the corresponding author. Microbiome relative abundance is included as supplement material (Supplementary file 3).

## Abbreviations

Css: Average steady state continuous IV infusion concentrations
CIs: Confidence intervals
CL: Clearance
CLR: Centered log-ratio
COV: Covariate
CYP: Cytochrome P450
EBEs: Empirical Bayes estimates
eGFR: Estimated glomerular function rate
F: Oral bioavailability
FFEM: Full fixed effect modeling
GvHD: Graft-versus-host disease
HCT: Allogeneic hematopoietic cell transplant
IIV: Interindividual variability
LASSO: Least Absolute Shrinkage and Selection Operator
OFV: Objective function value
pcVPC: Prediction-corrected visual predictive checks
PGP: P-glycoprotein protein
POR: P450 oxidoreductase
PsN: Perl-speaks-NONMEM
RUV: Residual unexplained variability
SCM: Stepwise covariate modeling
SIR: Sampling-importance-resampling
TAC: Tacrolimus
