# Supplementary material for: Identification of Microbiome Associations with Tacrolimus Pharmacokinetics in Adult Hematopoietic Cell Transplantation Using Population Pharmacokinetic and Machine Learning
Source: Pharm Res. 2026 Jul 20;43(9):3125–44. doi: 10.1007/s11095-026-04152-x (PMC13642457; doi:10.1007/s11095-026-04152-x)
Supplement: Supplementary file 1 [file 11095_2026_4152_MOESM2_ESM.pdf]

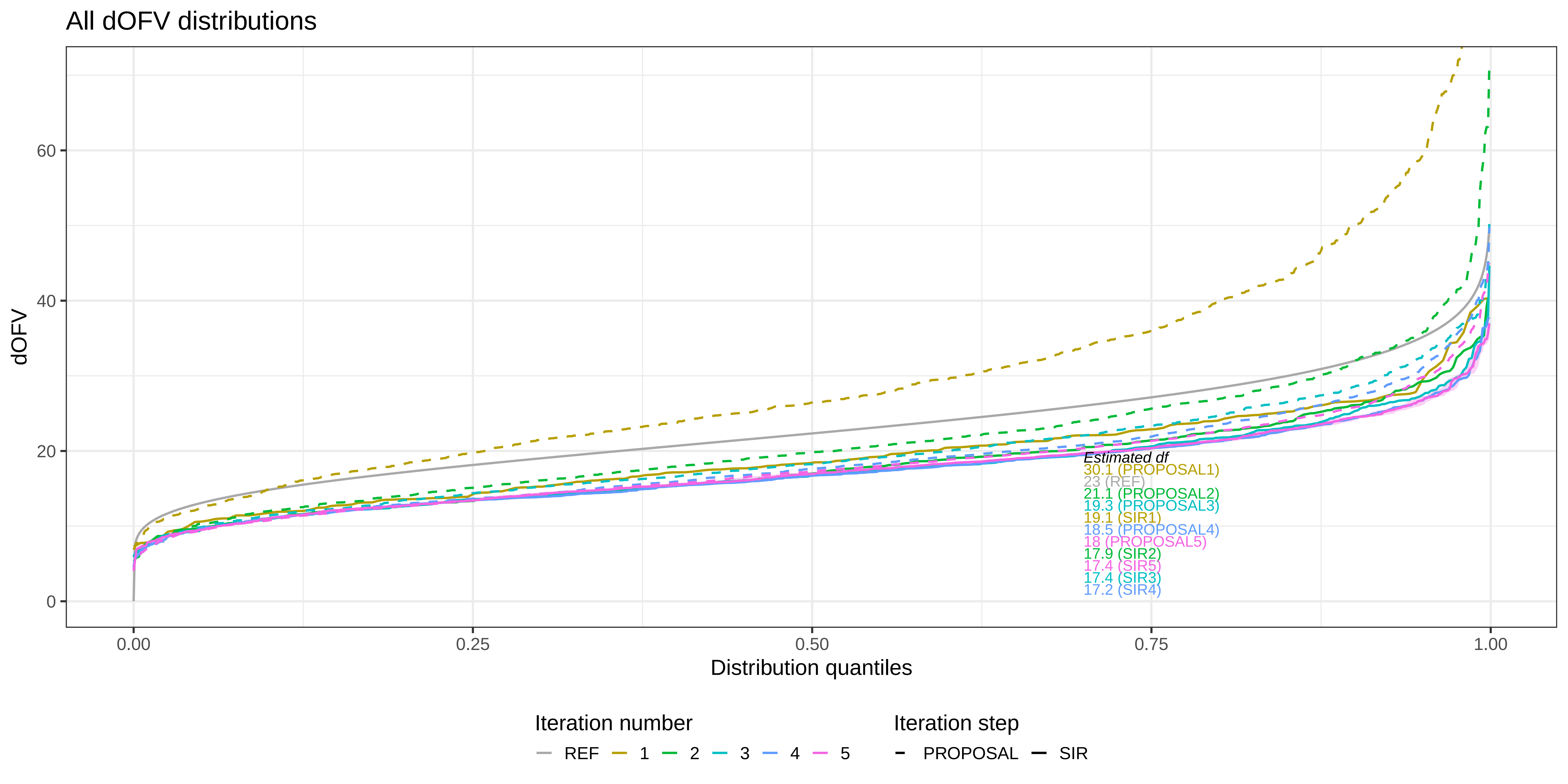
All dOFV distributions
dOFV
0
20
40
60
0.00
0.25
0.50
0.75
1.00
Distribution quantiles
Estimated df
30.1 (PROPOSAL1)
23 (REF)
21.1 (PROPOSAL2)
19.3 (PROPOSAL3)
19.1 (SIR1)
18.5 (PROPOSAL4)
18 (PROPOSAL5)
17.9 (SIR2)
17.4 (SIR5)
17.4 (SIR3)
17.2 (SIR4)
Iteration number
REF
1
2
3
4
5
Iteration step
PROPOSAL
SIR

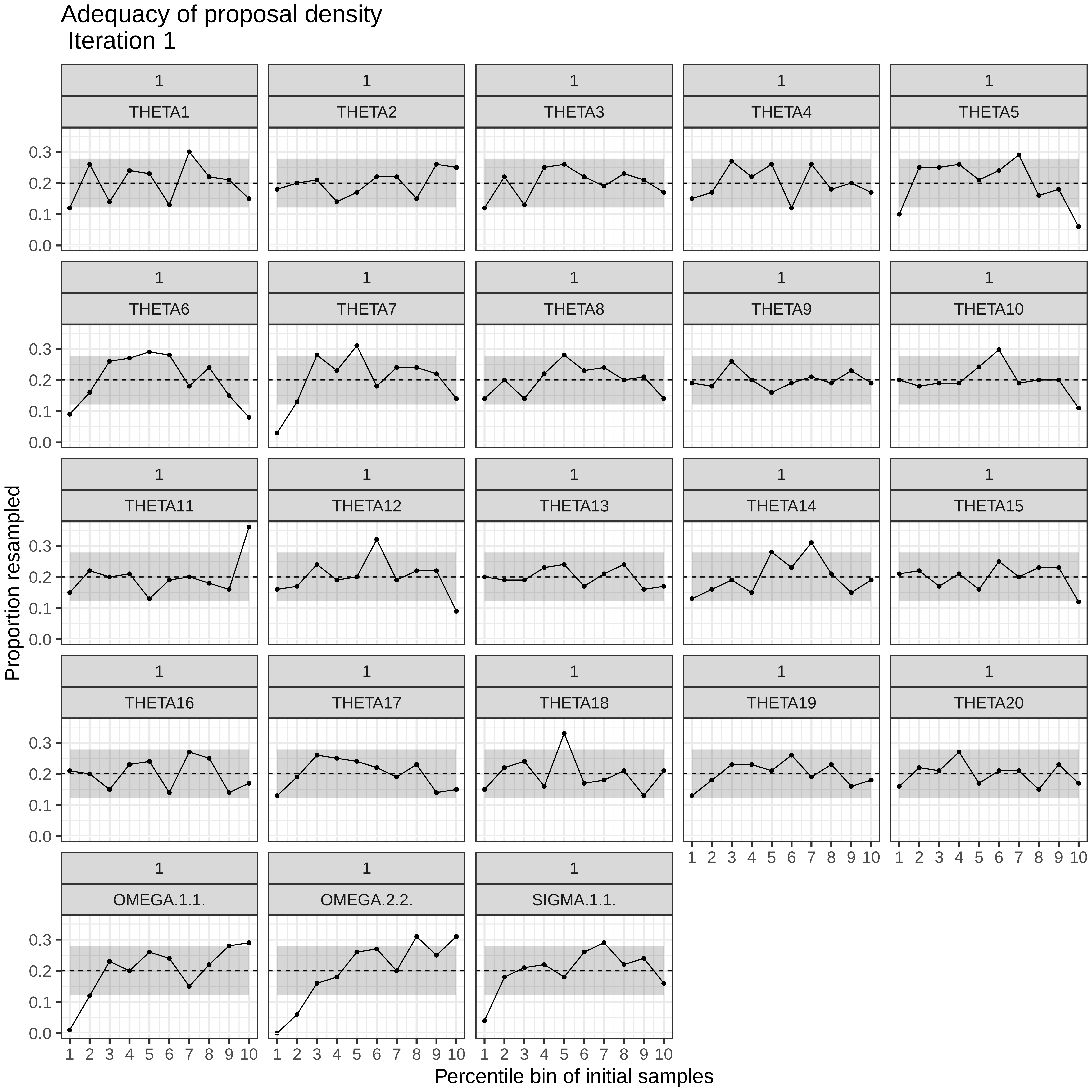

Adequacy of proposal density
Iteration 1
THETA1
THETA2
THETA3
THETA4
THETA5
THETA6
THETA7
THETA8
THETA9
THETA10
THETA11
THETA12
THETA13
THETA14
THETA15
THETA16
THETA17
THETA18
THETA19
THETA20
OMEGA.1.1.
OMEGA.2.2.
SIGMA.1.1.
Proportion resampled
Percentile bin of initial samples

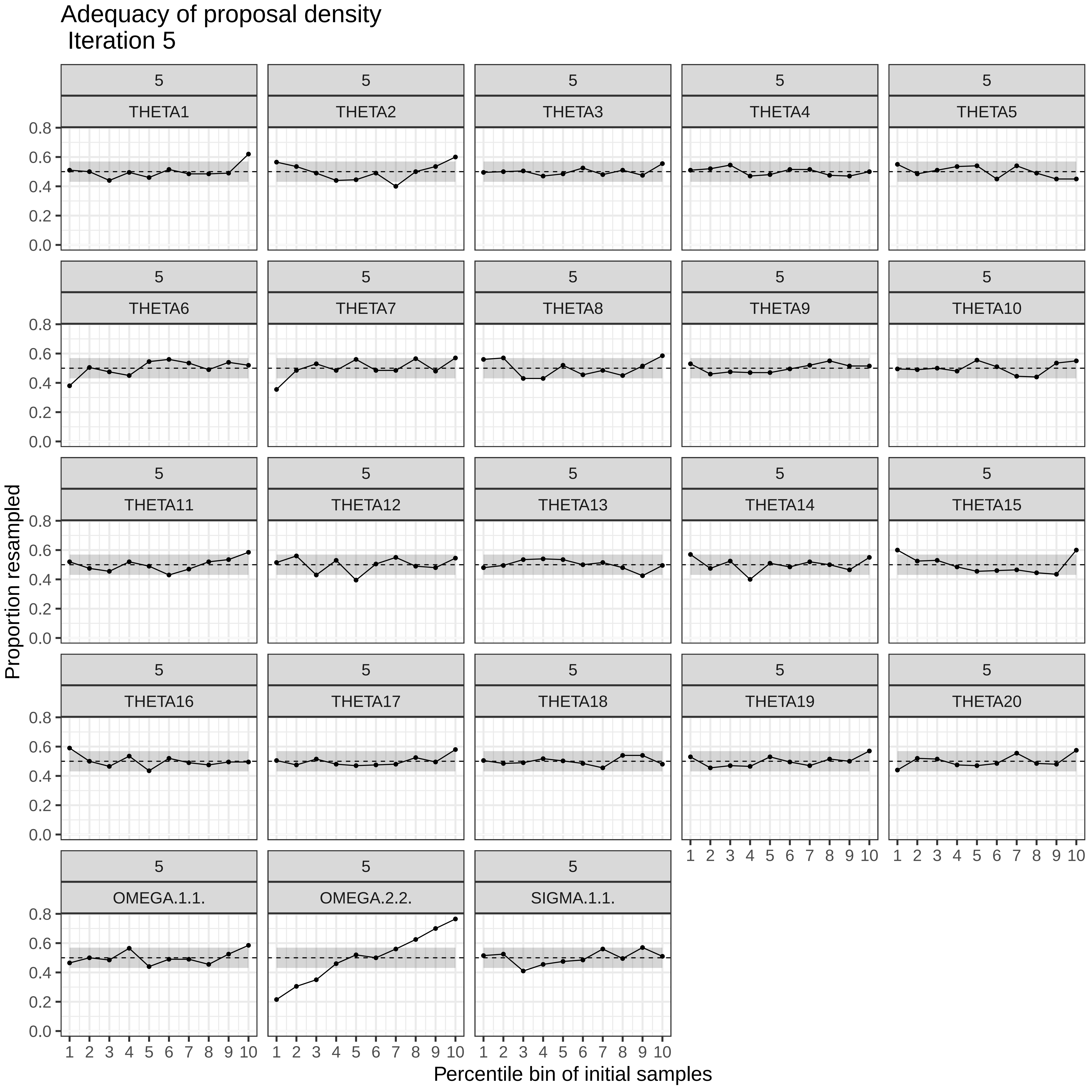
Adequacy of proposal density
Iteration 5
Proportion resampled
Percentile bin of initial samples
5
THETA1
THETA2
THETA3
THETA4
THETA5
THETA6
THETA7
THETA8
THETA9
THETA10
THETA11
THETA12
THETA13
THETA14
THETA15
THETA16
THETA17
THETA18
THETA19
THETA20
OMEGA.1.1.
OMEGA.2.2.
SIGMA.1.1.
0.0
0.2
0.4
0.6
0.8
1
2
3
4
5
6
7
8
9
10

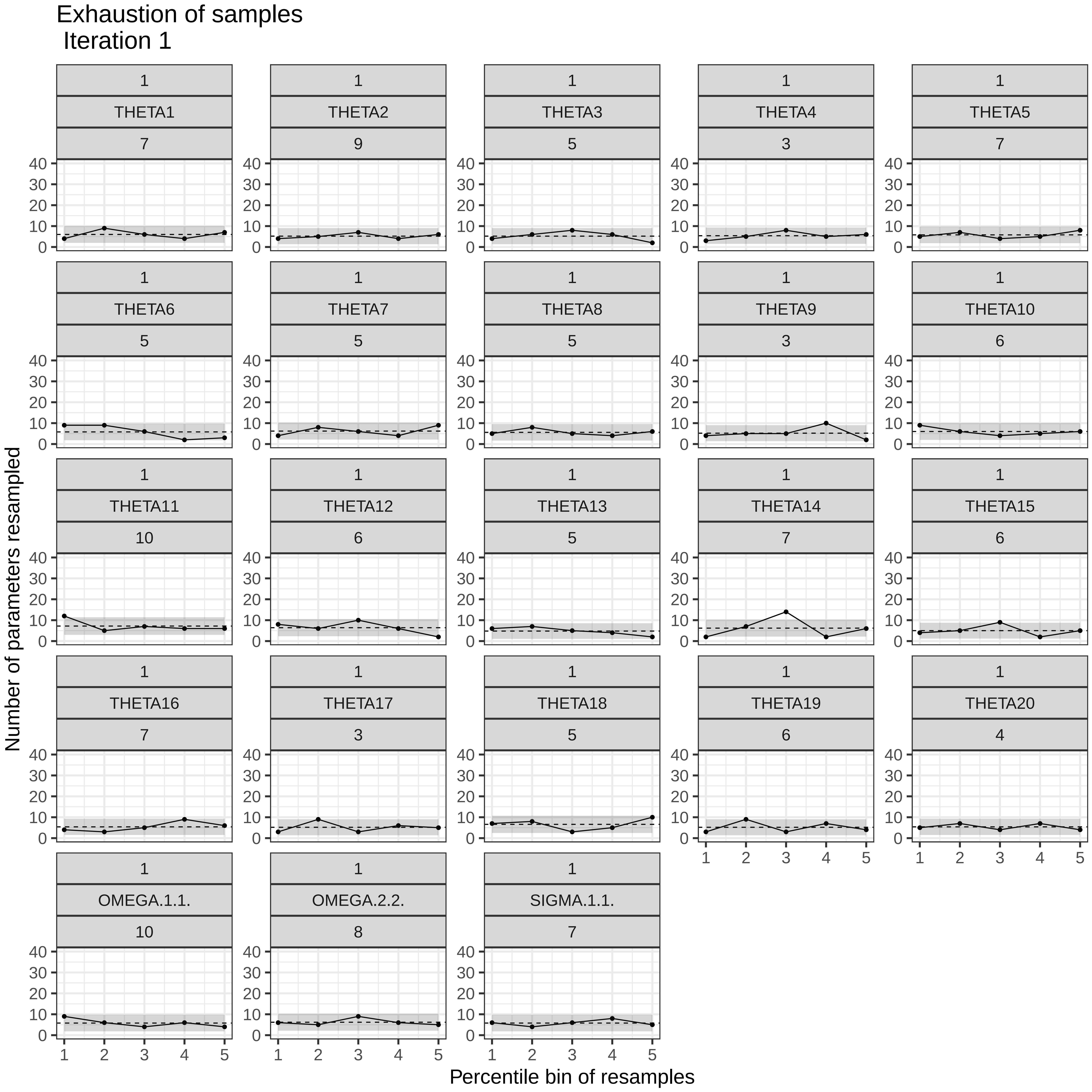
Exhaustion of samples
Iteration 1
1
THETA1
7
1
THETA2
9
1
THETA3
5
1
THETA4
3
1
THETA5
7
1
THETA6
5
1
THETA7
5
1
THETA8
5
1
THETA9
3
1
THETA10
6
1
THETA11
10
1
THETA12
6
1
THETA13
5
1
THETA14
7
1
THETA15
6
1
THETA16
7
1
THETA17
3
1
THETA18
5
1
THETA19
6
1
THETA20
4
1
OMEGA.1.1.
10
1
OMEGA.2.2.
8
1
SIGMA.1.1.
7
0
10
20
30
40
1
2
3
4
5
Number of parameters resampled
Percentile bin of resamples

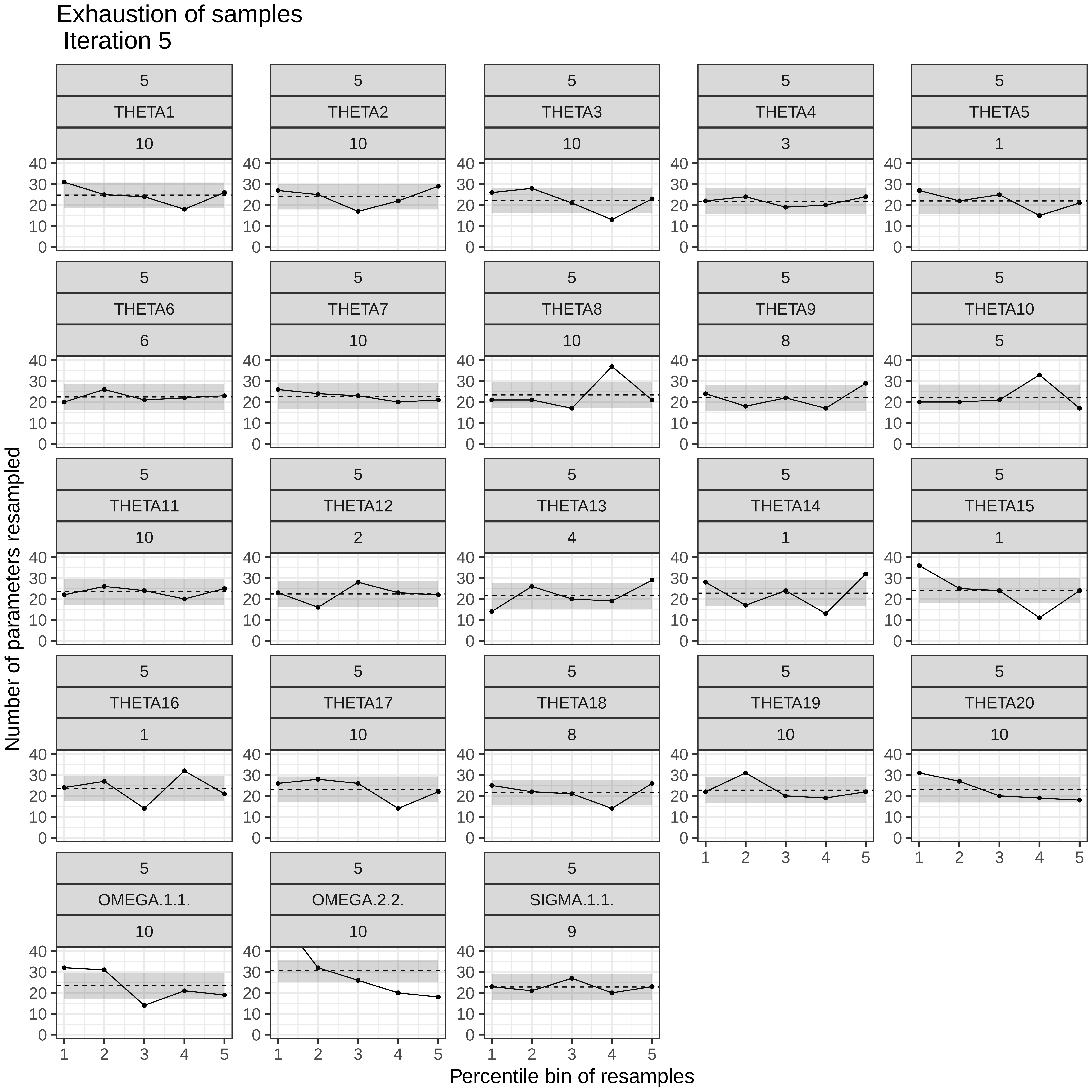
Exhaustion of samples
Iteration 5
5 THETA1 10
5 THETA2 10
5 THETA3 10
5 THETA4 3
5 THETA5 1
5 THETA6 6
5 THETA7 10
5 THETA8 10
5 THETA9 8
5 THETA10 5
5 THETA11 10
5 THETA12 2
5 THETA13 4
5 THETA14 1
5 THETA15 1
5 THETA16 1
5 THETA17 10
5 THETA18 8
5 THETA19 10
5 THETA20 10
5 OMEGA.1.1. 10
5 OMEGA.2.2. 10
5 SIGMA.1.1. 9
0 10 20 30 40
1 2 3 4 5
Number of parameters resampled
Percentile bin of resamples

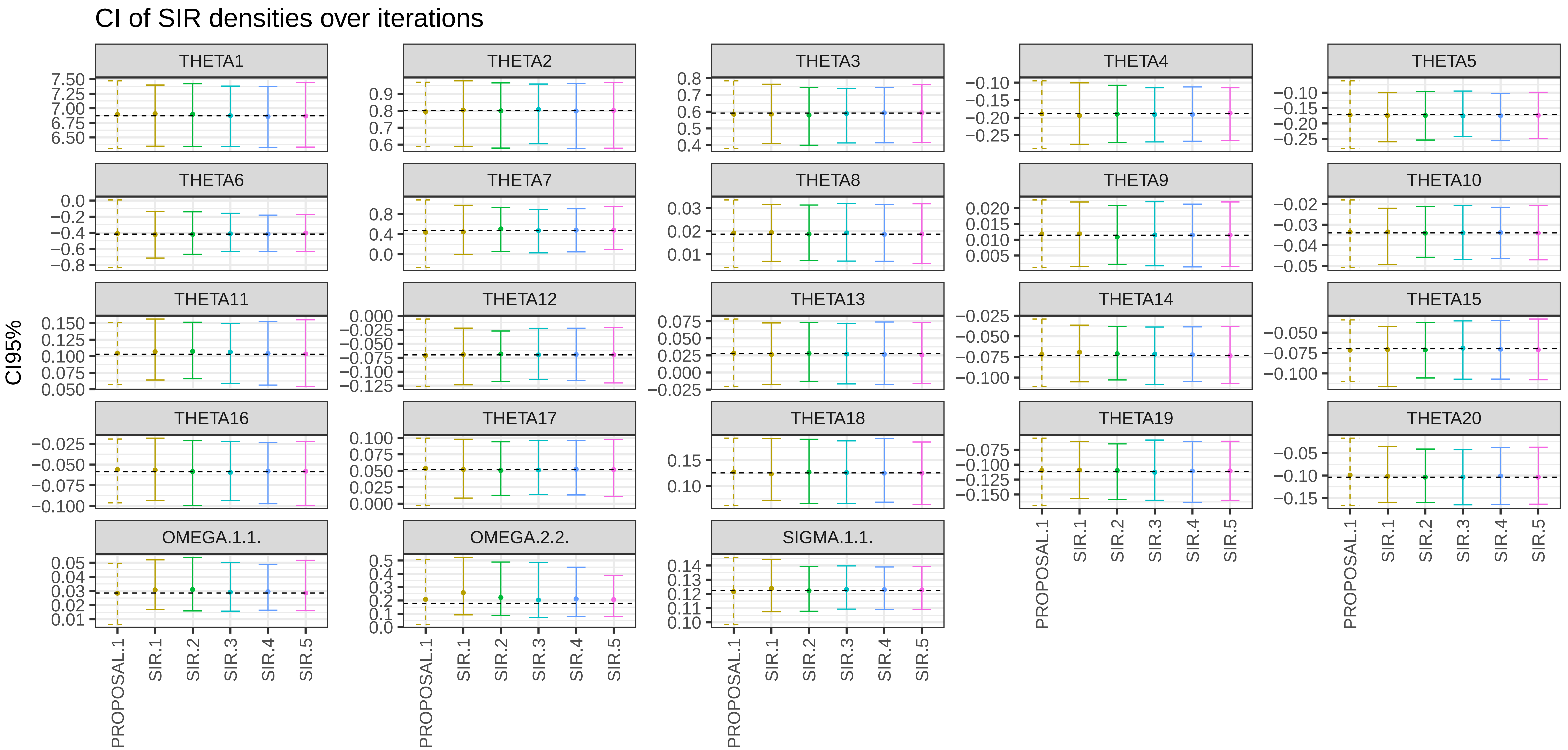
CI of SIR densities over iterations
CI95%
THETA1
THETA2
THETA3
THETA4
THETA5
THETA6
THETA7
THETA8
THETA9
THETA10
THETA11
THETA12
THETA13
THETA14
THETA15
THETA16
THETA17
THETA18
THETA19
THETA20
OMEGA.1.1.
OMEGA.2.2.
SIGMA.1.1.
PROPOSAL.1
SIR.1
SIR.2
SIR.3
SIR.4
SIR.5
