# Supplementary material for: Identification of Microbiome Associations with Tacrolimus Pharmacokinetics in Adult Hematopoietic Cell Transplantation Using Population Pharmacokinetic and Machine Learning
Source: Pharm Res. 2026 Jul 20;43(9):3125–44. doi: 10.1007/s11095-026-04152-x (PMC13642457; doi:10.1007/s11095-026-04152-x)
Supplement: Supplementary file 2 [file 11095_2026_4152_MOESM1_ESM.docx]

**Supplement Figure (1). Correlation between the CLR-transformed relative abundance of the top 32 bacterial species.** The correlation between bacterial species was tested using Spearman correlation.

**Supplement Figure (2). Volcano plot for differential analysis represents the effect size and significance level in samples collected before or at first oral TAC steady state trough (n=28) and samples collected after first oral TAC steady state trough (n=16).** MaAsLin 3 was used to analyze differential analysis. Species with at least 25% prevalence were included in the analysis and abundance models were only tested. Dashed line on Y-axis represents significance level at false discovery rate at 10%.

**A)**

**B)**

**Supplement Figure (3). Alpha diversity box plot (plot A) and volcano plot (plot B) showing effect size and statistical significance of a differential abundance analysis conducting in a subgroup of patients (n=35) with both day 14 and day 28 stool samples.** Plot A shows the difference in the alpha diversity indices (Shannon and inverse Simpson). Plot B shows the volcano plot of the differential abundance analyses which were performed using MaAsLin 3. Participant ID was included as a random effect to account for correlation between repeated samples. Species with at least 25% prevalence were included in the analysis and abundance models were only tested. Dashed line on Y-axis represents significance level at false discovery rate at 10%. Samples were not different after multiple testing correction.

**Supplement Figure (4). Box plot represents trend between CYP3A5*3 genotype and dose-adjusted TAC concentrations.**

**Supplement Figure (5). Importance plot of the LASSO regression models showing top predictive species.** Species with vertical bars are the ones predictive of tacrolimus pharmacokinetic parameters and included in the population pharmacokinetic model. Species not picked up by LASSO regression has no value on the x-axis and excluded from the analysis.

**Supplement Figure (6). Variance inflation factor analysis for the identified top predictive bacterial species.**

**Supplement Figure (7). Correlation between inter-individual variability in TAC clearance “CL” and bioavailability “F” and microbiome alpha-diversity.**

The inter-individual variability in CL (ETA 1) and F (ETA 2), after including the clinical factors and pharmacogenomics in the base model, were tested against the microbiome alpha diversity indices (Shannon and inverse Simpson) to identify trends using Pearson-correlation.

**Supplement Figure (8). Final model goodness-of-fit diagnostic plots.**

CWRES = conditional weighted residuals

**Supplement Figure (9). Forest plot of microbiome effects on tacrolimus clearance.**

This forest plot shows the effect of varying microbiome relative abundances on the TAC clearance (CL) at steady state with one microbiome altered at a time while fixing the other covariates at their median value. The x-axis is the fraction of simulated TAC CL relative to the CL at reference values and their 90% confidence interval (reference values are oral daily dose = 3 mg/day, median centered-log ratio (CLR)-transformed relative abundance, CYP3A5*3/*3, without concomitant medication use). The gray shaded area represents a 80-125% variation from the reference TAC CL. The vertical dashed line represents the null value at the median CLR-transformed relative abundance. The y-axis is the CLR-transformed relative abundance values ranked at 5th, 25th, 75th and 95th percentiles. If the fraction and its entire 90% CI is outside of the gray shaded area, the simulated effect of the microbiome covariate value was considered clinically relevant. If the 90% CI crosses the boundary between clinically relevant and irrelevant effects, the covariate clinically relevancy is uncertain.

**Supplement Figure (10). Forest plot of microbiome effects on tacrolimus bioavailability.**

This forest plot shows the effect of varying microbiome relative abundances on the oral TAC bioavailability (F) at steady state with one microbiome altered at a time while fixing the other covariates at their median value. The x-axis is the fraction of simulated TAC F relative to the F at reference values and their 90% confidence interval (reference values are oral daily dose = 3 mg/day, median centered-log ratio (CLR)-transformed relative abundance, CYP3A5*3/*3, without concomitant medication use). The gray shaded area represents a 80-125% variation from the reference TAC F. The vertical dashed line represents the null value at the median CLR-transformed relative abundance. The y-axis is the CLR-transformed relative abundance values ranked at 5th, 25th, 75th and 95th percentiles. If the fraction and its entire 90% CI is outside of the gray shaded area, the simulated effect of the microbiome covariate value was considered clinically relevant. If the 90% CI crosses the boundary between clinically relevant and irrelevant effects, the covariate clinically relevancy is uncertain.

**NONMEM control stream**

$SIZES	PD=-120

$PROB

$INPUT

$DATA

$SUBROUTINE

$PRED

;; F covariates

;;; F1Laxatives-DEFINITION START

IF(Laxatives.EQ.0) F1Laxatives = 0  ; Most common

IF(Laxatives.EQ.1) F1Laxatives = ( 0 + THETA(7))

;;; F1Laxatives-DEFINITION END

;;; F1Corticosteroids-DEFINITION START

IF(Corticosteroids.EQ.0) F1Corticosteroids = 0  ; Most common

IF(Corticosteroids.EQ.1) F1Corticosteroids = ( 0 + THETA(6))

;;; F1Corticosteroids-DEFINITION END

;;;T11 s__Ruthenibacterium_lactatiformans

Flactati = THETA(11)*(lactatiformans-(-0.6759))

;;;T12 s__Veillonella_atypica

Fatypica = THETA(12)*(atypica-(-1.7002))

;;;T13 s__Sellimonas_intestinalis

Fintesti = THETA(13)*(intestinalis-(-1.4625))

;;;T14 s__Enterococcus_faecium

Ffaecium = THETA(14)*(faecium-(-0.3043))

;;;T15 s__Bacteroides_uniformis

Funiformis = THETA(15)*(uniformis-(-0.7839))

;;;T16 s__Bacteroides_ovatus

Fovatus = THETA(16)*(ovatus-(-1.4625))

;;;T17 s__Limosilactobacillus_fermentum

Ffermentum  = THETA(17)*(fermentum -(-1.6278))

;;;T18 s__Eggerthella_lenta

Flenta = THETA(18)*(lenta-(-1.5057))

;;;T19 s__Blautia_caecimuris

Fcaecimuris = THETA(19)*(caecimuris-(-1.7002))

;;;T20 s__Streptococcus_salivarius

Fsalivarius = THETA(20)*(salivarius-(-1.3441))

;;;END microbime

;;; F1-RELATION START

F1COV=F1Corticosteroids+F1Laxatives+Flactati+Fatypica+Fintesti+Ffaecium+Funiformis+Fovatus+Ffermentum+Flenta+Fcaecimuris+Fsalivarius

;;; F1-RELATION END

;;; CL covariates

;;; CLeGFR_EPI_RF_grp-DEFINITION START

IF(eGFR_EPI_RF_grp.EQ.0) CLeGFR_EPI_RF_grp = 1  ; Most common

IF(eGFR_EPI_RF_grp.EQ.1) CLeGFR_EPI_RF_grp = ( 1 + THETA(5))

;;; CLeGFR_EPI_RF_grp-DEFINITION END

;;; CLVORICONAZOLE-DEFINITION START

IF(VORICONAZOLE.EQ.0) CLVORICONAZOLE = 1  ; Most common

IF(VORICONAZOLE.EQ.1) CLVORICONAZOLE = ( 1 + THETA(4))

;;; CLVORICONAZOLE-DEFINITION END

;;; CLCYP3A5_3_2-DEFINITION START

IF(CYP3A5_3_2.EQ.1) CLCYP3A5_3_2 = 1  ; Most common

IF(CYP3A5_3_2.EQ.0) CLCYP3A5_3_2 = ( 1 + THETA(3))

;;; CLCYP3A5_3_2-DEFINITION END

;; microbiome centered to the median

;;; CL s__Bacteroides_uniformis

CLuniformis = EXP(THETA(8)*(uniformis-(-0.7839)))

;;; CL s__Bacteroides_ovatus

CLovatus = EXP(THETA(9)*(ovatus-(-1.4625)))

;;; CL s__Limosilactobacillus_fermentum

CLfermentum = EXP(THETA(10)*(fermentum-(-1.6278)))

;;; END microbime

;;; CL-RELATION START

CLCOV=CLCYP3A5_3_2*CLVORICONAZOLE*CLeGFR_EPI_RF_grp*CLuniformis*CLovatus*CLfermentum

;;; CL-RELATION END

TVCL =THETA(1)

TVCL = CLCOV*TVCL

CL= TVCL*EXP(ETA(1)) ; TAC_CL_L_hr

TVF1 = THETA(2)

TVF1 = F1COV+TVF1

logitF1 = TVF1 + ETA(2)

IF (ROUTE.EQ.0) F1=1

IF (ROUTE.EQ.1) F1= EXP(logitF1)/(EXP(logitF1)+1) ; TAC_oral_F(logit transformed)[fix it between =0,1]

CSS = F1*RATE*1e3 / CL

F= CSS

IPRED=F

W = SQRT(IPRED**2*SIGMA(1,1))

DEL=0.001

IRES=DV-IPRED

IWRES=IRES/(W+DEL)

Y = F + F*ERR(1)

$THETA

(0, 6.9101)         ; T1 TVCL total

(-2, 0.59240, 2) ; T2 TVF oral

(-1, 0.57532, 5) ; CLCYP3A5_3_21

(-1, -0.20317, 5) ; CLVORICONAZOLE1

(-1, -0.16902, 5) ; CLeGFR_EPI_RF_grp1

(-2, -0.44743, 2) ; F1Corticosteroids1

(-2, 0.52704, 2) ; F1Laxatives1

(-2, 0.020259, 2) ;T8 CL_Bacteroides_uniformis

(-2, 0.011339, 2) ;T9 CL_Bacteroides_ovatus

(-2, -0.034464, 2) ;T10 CL_Limosilactobacillus_fermentum

(-2, 0.11965, 2) ;T11 F_Ruthenibacterium_lactatiformans

(-2, 0.050486, 2) ;T12 F_Veillonella_atypica

(-2, 0.050774, 2) ;T13 F_Sellimonas_intestinalis

(-2, -0.060493, 2) ;T14 F_Enterococcus_faecium

(-2, -0.067766, 2) ;T15 F_Bacteroides_uniformis

(-2, -0.059150, 2) ;T16 F_Bacteroides_ovatus

(-2, 0.055190, 2) ;T17 F_Limosilactobacillus_fermentum

(-2, 0.13937, 2) ;T18 F_Eggerthella_lenta

(-2, -0.11461, 2) ;T19 F_Blautia_caecimuris

(-2, -0.072378, 2) ;T20 F_Streptococcus_salivarius

$OMEGA

0.026110    ; IIV[EXP] on CL

0.20796   ; IIV[EXP] on F

$SIGMA

0.12228          ;    ERR1[P]

$ESTIMATION METHOD=1 INTER NOABORT MAXEVAL=9999 PRINT=1 NSIG=3 SIGL=9

$COVARIANCE MATRIX=R PRINT=E UNCONDITIONAL

$TABLE ID TIME DV CL F1 IPRED CWRES IWRES ROUTE ONEHEADER NOPRINT FILE=sdtab1

$TABLE ID ETAS(1:LAST) CL F1 TIME ONEHEADER NOPRINT FILE=patab1 ; model parameters

$TABLE ID CYP3A5_3_2 VORICONAZOLE eGFR_EPI_RF_grp Corticosteroids Laxatives ONEHEADER NOPRINT FILE=catab1 ; categorical covariates

$TABLE ID lactatiformans intestinalis faecium uniformis ovatus fermentum lenta caecimuris salivarius atypica  ONEHEADER NOPRINT FILE=cotab1 ; continuous covariates
